# Using deep learning and large protein language models to predict protein–membrane interfaces of peripheral membrane proteins

**DOI:** 10.1093/bioadv/vbae078

**Published:** 2024-05-28

**Authors:** Dimitra Paranou, Alexios Chatzigoulas, Zoe Cournia

**Affiliations:** Biomedical Research Foundation, Academy of Athens, Athens 11527, Greece; Department of Informatics and Telecommunications, National and Kapodistrian University of Athens, Athens 15784, Greece; Biomedical Research Foundation, Academy of Athens, Athens 11527, Greece; Biomedical Research Foundation, Academy of Athens, Athens 11527, Greece; Department of Informatics and Telecommunications, National and Kapodistrian University of Athens, Athens 15784, Greece

## Abstract

**Motivation:**

Characterizing interactions at the protein–membrane interface is crucial as abnormal peripheral protein–membrane attachment is involved in the onset of many diseases. However, a limiting factor in studying and understanding protein–membrane interactions is that the membrane-binding domains of peripheral membrane proteins (PMPs) are typically unknown. By applying artificial intelligence techniques in the context of natural language processing (NLP), the accuracy and prediction time for protein–membrane interface analysis can be significantly improved compared to existing methods. Here, we assess whether NLP and protein language models (pLMs) can be used to predict membrane-interacting amino acids for PMPs.

**Results:**

We utilize available experimental data and generate protein embeddings from two pLMs (ProtTrans and ESM) to train classifier models. Overall, the results demonstrate the first proof of concept study and the promising potential of using deep learning and pLMs to predict protein–membrane interfaces for PMPs faster, with similar accuracy, and without the need for 3D structural data compared to existing tools.

**Availability and implementation:**

The code is available at https://github.com/zoecournia/pLM-PMI. All data are available in the Supplementary material.

## 1 Introduction

Membrane proteins comprise 40% of all protein pharmaceutical targets. They can be divided into transmembrane proteins, which are integrated in the membrane; peripheral membrane proteins (PMPs), which are temporarily and non-covalently attached to the membrane surface or to integral proteins; and lipid-anchored proteins, which bind to the membrane through a lipid molecule that is covalently linked to a specific amino acid residue in the protein (Boes *et al.* 2021). PMPs are responsible for a variety of biological functions, including cell signaling and recognition, membrane trafficking, cell division, and cell shape maintainance (Monje-Galvan and Klauda 2016). Through a series of distinct mechanisms, PMPs are drawn to and interact with cellular membranes with one or more domains driving the protein–membrane binding (Whited and Johs 2015, Boes *et al.* 2021). The abnormal attachment of PMPs to the membrane is involved in the deregulation of their function, which may result in the development of human diseases (cancer, diabetes, and others) (Monje-Galvan and Klauda 2016, Boes *et al.* 2021). Recent years have seen a rise in the study of PMPs due to their significance in numerous physiological functions, their implication in disease, and their emergence as therapeutic targets ([Bibr vbae078-B11][Bibr vbae078-B26], Zukowska *et al.* 2015, Knight *et al.* 2018, Vanhaesebroeck *et al.* 2022). However, the development of drugs targeting PMPs has been limited due to the complexity of their interactions with cellular membranes and because the membrane-binding domains of PMPs are typically unknown (Chatzigoulas and Cournia 2022a).

Several tools that detect protein–membrane regions have recently appeared in the literature (Lomize *et al.* 2012, 2022, Kufareva *et al.* 2014, Chatzigoulas and Cournia 2022a, van Hilten et al. 2024). In our previous work, we presented an ensemble machine learning (ML) classifier model called DREAMM (Chatzigoulas and Cournia 2022a, 2022b) that predicts protein–membrane interfaces of PMPs and displays similar or better accuracy compared to other methods that predict protein–membrane interfaces from the 3D protein structure, such as PPM (Lomize *et al.* 2022, 2012) and MODA (Kufareva *et al.* 2014). DREAMM is integrated into a computational drug design workflow targeting protein–membrane interfaces and it is offered as a free web-server (https://dreamm.ni4os.eu/) (Chatzigoulas and Cournia 2022a). More recently, a transformer neural network (NN) model called PMIpred was trained in coarse-grained molecular simulation data of over 50 000 peptides on a neutral model membrane (van Hilten *et al.* 2023). Using the attention mechanism of transformers, PMIpred was trained using peptide sequences as input and their relative membrane-binding free energy as output. PMIpred predicts the relative membrane-binding free energy for any given amino acid sequence and the membrane-interacting amino acids with an accuracy comparable to DREAMM, PPM, and MODA. One of the limitations of previous ML approaches is that they are time-consuming, requiring several minutes to hours to complete the calculation depending on the protein size. For example, in DREAMM, the time-consuming part is the feature extraction process, which accounts for more than 99% of the total prediction time.

Large language models (LLMs) are a category of foundation models trained on enormous amounts of data that can understand and generate natural language and other types of content to perform a wide range of tasks. LLMs based on deep learning techniques utilize the transformer architecture and its attention mechanism (Vaswani *et al.* 2017) to process and generate natural language efficiently. The enhancement of LLMs through transfer learning and fine-tuning for specific downstream tasks have inspired the generation of pre-trained LLMs that are now used by researchers to solve downstream tasks and achieve faster times while keeping similar accuracy outcomes (Rao *et al.* 2019, 2020, 2021). In this context, protein language models (pLMs) that are trained on vast datasets of protein sequences spanning the evolutionary tree of life have the potential to learn patterns in protein sequences across evolution, thus facilitating progress in diverse applications such as direct inference of full atomic-level protein structure prediction from its primary sequence (Lin *et al.* 2023).

Herein, we train classifier models using pLMs to predict protein–membrane interfaces; the approach significantly reduces prediction times and achieves improved accuracy compared to existing tools, albeit for proteins that belong in the same superfamilies that are present in the training set. We thus provide the first proof of concept that deep learning and pLMs have a promising potential to predict protein-membrane interfaces for PMPs faster and with similar accuracy compared to existing tools. We find that the prediction quality is dependent on the original training set and on whether the input protein sequences are structurally-resolved; as data for PMPs are scarce, we discuss the strengths and limitations of such models given the limited protein–membrane interface experimental data.

## 2 Methods

### 2.1 Data collection and data preparation

For dataset construction, two publicly available PMP datasets were used. The first dataset was downloaded from Tubiana *et al.* (2022) (PePrMInt dataset (2022)), which contains nine protein domains binding transiently to the membrane, namely Annexin, C1, C2, discoidin C2, PH, PX, PLA, PLC/D, and START. This dataset has been generated by first annotating the membrane-binding sites in each superfamily using experimental data from the literature and then transferring that annotation to other domains in the same superfamilies based on structural alignment. It includes 1963 PDB IDs (674 UniProt IDs—one UniProt ID can correspond to multiple PDB IDs) with interfacial binding sites (IBS)—IBS is the nomenclature used in PePrMInt to describe the amino acids that interact with the membrane.

The second collection of PMPs was retrieved from the DREAMM project and consists of 65 proteins with known 3D structures and experimentally known membrane-penetrating amino acids or membrane-interacting regions (Chatzigoulas and Cournia 2022a, 2022b). The difference between the two PMP datasets is that the PePrMInt dataset defines the IBS as the whole secondary structural element of the experimentally membrane-interacting amino acids (e.g. the whole loop or helix), while the DREAMM dataset contains only the hydrophobic membrane-penetrating amino acids. The two datasets have 30 PDB IDs in common. To create the same reference point between the two datasets, the UniProt IDs were kept from the PePrMInt dataset, and for the DREAMM proteins the PDB IDs were corresponded to UniProt IDs.

Then, for a total of 709 unique UniProt IDs, the FASTA sequences were retrieved from the UniProt database (UniProt Consortium 2019) and the sequence similarities were calculated by clustering the protein sequences to avoid a biased dataset because the proteins in the PePrMInt dataset belong to nine superfamilies. Utilizing the CD-HIT Suite (Fu *et al.* 2012) the sequence identity cutoff was set to 40%, which produced 443 protein clusters. For each cluster, CD-HIT provided a representative sequence, which is the largest sequence of the cluster. From the clustering results, only the representatives of each cluster were kept. The corresponding PDB code of every representative sequence was fetched from the Protein Data Bank (PDB) (Berman *et al.* 2000) and was aligned with the corresponding UniProt sequences (for more information about the alignment see the Supplementary material). In those cases, where the PDB protein is a multimer, only the chain that corresponds to the sequence of the related UniProt ID was kept. For the proteins that were common between the PePrMInt and the DREAMM datasets, the annotation of membrane association was performed according to the IBS definition of the PePrMInt dataset. Then, the IBS amino acids of UniProt sequences were updated to match the annotation of each corresponding aligned PDB sequence (for more information see Supplementary material and Supplementary Fig. S1). Finally, a collection of 443 (411 from PePrMInt and 32 from DREAMM) peripheral membrane proteins and 325 943 amino acids was created. The pipeline for data preparation with UniProt sequences is displayed in Supplementary Fig. S2.

Moreover, we generated a second PMP sequence collection using the same procedure as described above, but instead of using as input the UniProt sequences, we utilized only the sequence part that is structurally resolved in their respective PDB entries. As with the UniProt sequence collection, the protein sequences from PePrMInt and DREAMM were used. The same data preprocessing steps were followed, including sequence clustering using CD-HIT and keeping representative protein sequences. Processing these sequences with CD-HIT resulted in 450 clusters with 418 proteins from PePrMInt, 32 proteins from DREAMM, and 76 084 amino acids in total. The pipeline for data preparation with PDB sequences is displayed in Supplementary Fig. S3.

### 2.2 Feature extraction using protein language models

To extract features, we utilized two of the most widely used pLMs, ProtTrans (Elnaggar *et al.* 2022) and the evolutionary scale modeling (ESM) (Lin *et al.* 2023) (for more information about these pLMs see the Supplementary material). These pLMs have been trained using sequence databases such as UniRef50 (Suzek *et al.* 2015) and BFD (Steinegger *et al.* 2019), which include millions of protein sequences and billions of amino acids. These models can be used for several downstream tasks such as secondary structure prediction, discovery of genetic variations, capturing of biophysical features of amino acids, prediction of protein subcellular localization, and others (Elnaggar *et al.* 2022, Lin *et al.* 2023). The last hidden layer of the encoder part of pLMs is commonly referred to as “contextual embeddings” (hereafter called embeddings), which is a continuous vector representation that captures the contextual information of the input sequence and can be further used for downstream prediction tasks. These vectors encode the structural and functional properties of a protein and serve as features for making predictions about protein structure, function, and other properties based on its amino acid sequence.

Here, we employ the prot_t5_xl_half_uniref50-enc model from ProtTrans and the esm2_t33_650M_UR50D from ESM (hereafter referred to as ProtTrans and ESM) (due to memory limitations, we could not utilize larger pLMs such as the esm2_t36_3B_UR50D or the esm2_t48_15B_UR50D). ProtTrans produced embedding vectors with a size of sequence_length×1024, while ESM yields embeddings of size sequence_length×1280. The procedure for extracting this information was highly efficient, requiring only ∼10 s for ProtTrans and ∼3 s for ESM for a medium-length protein (up to 500 amino acids) in a MacBook Pro with M1, 10 cores, and 16 GB RAM.

For the collection of the 443 UniProt sequences (411 from PePrMInt and 32 from DREAMM), we generated embeddings and utilized them as features. Then, using 410 PePrMInt protein sequences, we created two datasets (for one PePrMInt protein, the IBS from the PDB sequence did not correspond to any amino acid of the UniProt sequence after alignment and thus, it could not be used for training). The first dataset contained ProtTrans embeddings and the second dataset contained ESM embeddings. Then, we converted the amino acid feature column from categorical to numerical features, by using one-hot encoding, a technique that transforms each unique value in a categorical feature into a new binary feature (part of the final dataset with the ProtTrans embeddings is seen in Supplementary Table S1). Finally, each dataset was split into a training set (∼80% of the dataset—328 proteins with 297 295 amino acids), a validation set (∼10%—41 proteins with 5967 amino acids), and a test set (∼10%—41 proteins with 7776 amino acids) (Supplementary Table S2). Furthermore, an extra test set was created containing the 32 DREAMM proteins and the one protein from the PePrMInt that could not be used for training (see comment above), resulting in a final extra test set of 33 proteins with 14 905 amino acids, Supplementary Table S2. These 33 proteins of the extra test set were evaluated qualitatively because they lack the IBS annotation of the PePrMInt dataset; instead experimentally identified membrane-interacting regions or a few known membrane-penetrating amino acids are annotated.

For the collection of the 450 PDB sequences (418 from PePrMInt and 32 from DREAMM), the same feature extraction procedure was used. Using the PDB sequences of the 418 PePrMInt proteins, we again created two datasets. The first dataset contained ProtTrans embeddings and the second dataset contained ESM embeddings. Each dataset was split into a training set (∼80% of the dataset—334 proteins with 53 149 amino acids), a validation set (∼10%—42 proteins with 6616 amino acids), and a test set (∼10%—42 proteins with 6875 amino acids) (Supplementary Table S3). Furthermore, an extra test set was created containing the 32 DREAMM proteins with 9444 amino acids (Supplementary Table S3). Again, the 32 proteins of the extra test set were evaluated qualitatively because of the lack of an IBS annotation.

**Figure 1.**
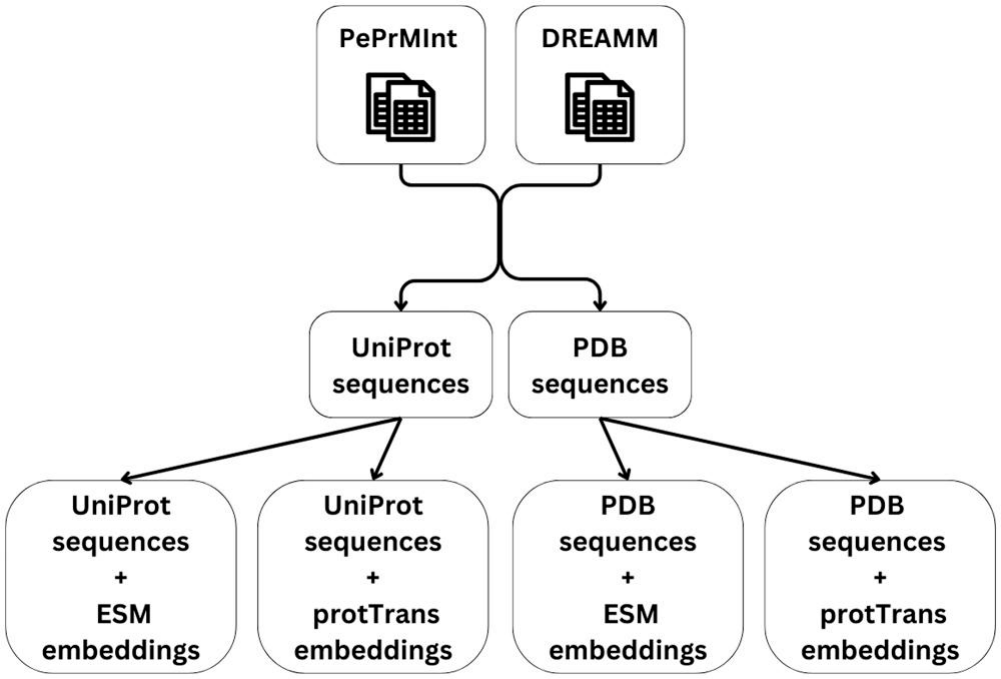
The generated dataset collections with UniProt and PDB sequences and the embeddings from the ESM and protTrans protein language models.

In Fig. 1, we summarize the datasets that were generated using the UniProt and PDB sequences and the ESM and ProtTrans embeddings as features.

### 2.3 Machine learning classifiers

To define which amino acids are at the protein–membrane interface, ML classifiers were used to predict the class (membrane-interacting or non-membrane-interacting) of a given amino acid. In this study, five ML classifiers were trained: eXtreme Gradient Boosting (XGBoost) classifier (Chen and Guestrin 2016), Light Gradient-Boosting Machine (LGBM) classifier (Ke *et al.* 2017), Balanced Random Forest classifier (Lemaître *et al.* 2017), and two NNs (Single-Layer Perceptron and Multi-Layer Perceptron) from the Keras library (Chollet 2015). These algorithms were selected due to their extensive use and successful performance across various tasks, as well as their ability to effectively handle imbalanced datasets such as ours (only 3% of the total number of amino acids belong to the membrane-interacting class). Herein, the approach of model penalization was used, which biases the model to emphasize the minority class by imposing a weighted cost when a sample is predicted incorrectly.

To choose the most accurate model for predicting membrane-interacting amino acids, we utilized the $F_{1}$ score and the Matthews correlation coefficient (MCC) as evaluation metrics, both of which are based on the confusion matrix (Supplementary material and Supplementary Fig. S4). Subsequently, hyperparameter optimization was performed on all classifiers using the Optuna framework (Akiba *et al.* 2019), to effectively separate the two classes based on the validation set. The Optuna library employs the Tree-structured Parzen Estimator algorithm, a Bayesian optimization (Snoek *et al.* 2012) technique that seeks to find an optimal set of hyperparameters by balancing exploration and exploitation using a probabilistic model based on Bayesian statistics (Supplementary material and Supplementary Fig. S5). This approach is particularly advantageous for complex models with multiple hyperparameters, outperforming other methods such as grid search or random search, converging toward optimal solutions.

The optimization of the five ML classifiers was conducted on both datasets of UniProt sequences (ESM & ProtTrans, see Fig. 1), including the hyperparameter that handles the dataset imbalance. For XGBoost and LGBM, the scale_pos_weight parameter was explored, while for NNs, the class_weight parameter was tested in initial runs, but because it did not improve the performance, we did not finetune it further. Balanced random forest inherently addresses the imbalance issue without specific hyperparameter tuning. The optimization process involved an extensive search within a wide range of values for each hyperparameter set. Specifically, 200 trials were conducted for XGBoost, LGBM, and Balanced Random Forest, while 50 trials were performed for Single-Layer Perceptron and Multi-Layer Perceptron (MLP).

Similarly, for both datasets of PDB sequences (ESM & ProtTrans), we optimized the hyperparameters of the LGBM classifier by conducting 200 trials.

**Figure 2.**
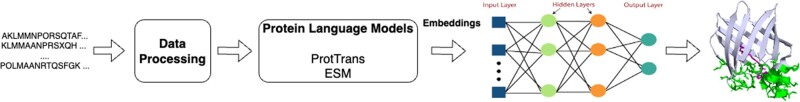
A schematic workflow of this study.

The general workflow of this study is represented in Fig. 2.

## 3 Results

### 3.1 Predicting protein–membrane interfaces of peripheral membrane proteins using pLMs

The classifiers with the best hyperparameters were first evaluated on the UniProt sequences of the test set, and the evaluation metrics are presented in Tables 1 and 2 for each dataset of UniProt sequences (Fig. 1). Detailed information on the search space and optimal hyperparameters for each model on each dataset can be found in Supplementary Table S4. The most promising classifier in both cases was the MLP (Supplementary Fig. S6), with the best model trained on the ProtTrans embeddings with a 0.68 $F_{1}$ score and 0.65 MCC.

**Table 1.** Model performance on the test set using ProtTrans embeddings of UniProt sequences with optimal hyperparameters determined by the Optuna framework.

Metric	XGBoost		LGBM		BalancedRandomForest		Single-layer perceptron		Multi-layer perceptron	
$F_{1}$ score	0.58		0.67		0.53		0.66		**0.68**	
MCC	0.53		0.63		0.47		0.63		**0.65**	
Confusion Matrix	**TP**	**FP**	**TP**	**FP**	**TP**	**FP**	**TP**	**FP**	**TP**	**FP**
	534	438	579	275	604	828	508	175	539	182
	**FN**	**TN**	**FN**	**TN**	**FN**	**TN**	**FN**	**TN**	**FN**	**TN**
	330	6474	285	6637	260	6084	356	6737	325	6730

The best score for each metric is highlighted in bold. The rows correspond to the actual classes and the columns to the predictions.

**Table 2.** Model performance on the test set using ESM embeddings of UniProt sequences with optimal hyperparameters determined by the Optuna framework.

Metric	XGBoost		LGBM		BalancedRandomForest		Single-layer perceptron		Multi-layer perceptron	
$F_{1}$ score	0.41		0.54		0.43		0.52		**0.58**	
MCC	0.38		0.48		0.35		0.52		**0.56**	
Confusion Matrix	**TP**	**FP**	**TP**	**FP**	**TP**	**FP**	**TP**	**FP**	**TP**	**FP**
	266	177	500	496	442	743	338	97	421	156
	**FN**	**TN**	**FN**	**TN**	**FN**	**TN**	**FN**	**TN**	**FN**	**TN**
	598	6735	364	6416	422	6169	526	6815	443	6756

The best score for each metric is highlighted in bold. The rows correspond to the actual classes and the columns to the predictions.

### 3.2 Visualizing predictions: comparative analysis of the most promising models

**Figure 3.**
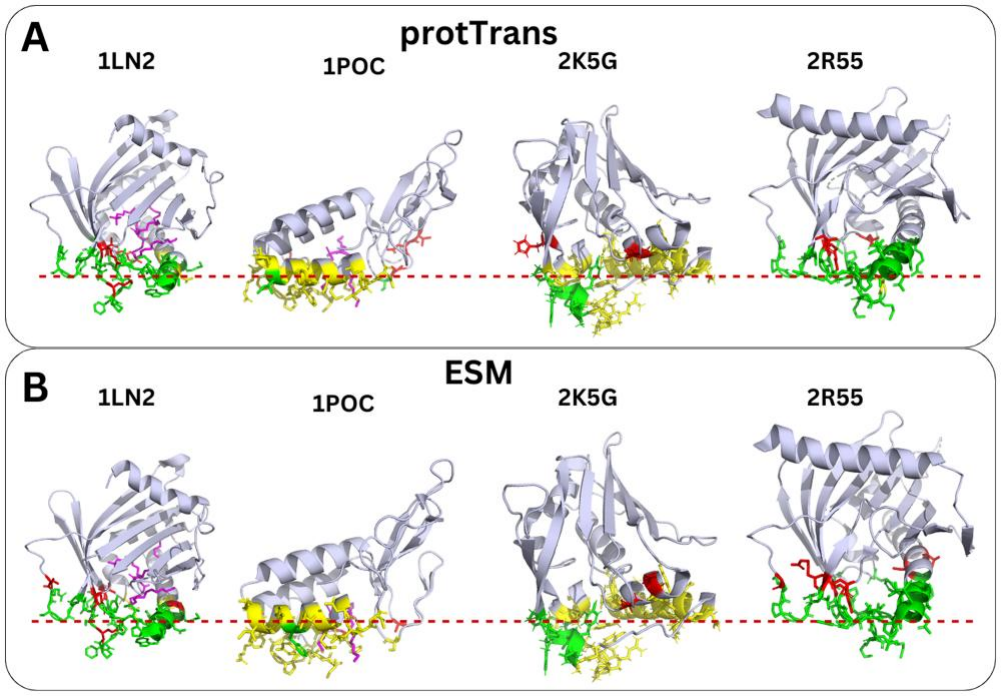
Four proteins of the test set with the predictions from the MLP models trained on (A) ProtTrans embeddings and (B) ESM embeddings. With green, we denote the TP, with yellow the FN, with red the FP, and with purple the ligands. The putative membrane plane is depicted as a red–dotted line and is aligned with the principal axis of all amino acids labeled as IBS in Tubiana *et al.* (2022).

To gain insights into the classifier model predictions, we visualized the results and inspected which amino acids are labeled as false positive (FP), false negative (FN), and true positive (TP) using PyMOL (DeLano 2020). In Fig. 3, snapshots of the predictions of four randomly-selected proteins from the test set [1LN2 (Roderick *et al.* 2002), 1POC (White *et al.* 1990), 2K5G (RCSB PDB - 2K5G), 2R55 (Thorsell *et al.* 2011)] using the two MLP models trained on the ESM and ProtTrans embeddings are shown. It is evident that MLP models accurately predict membrane-interacting amino acids, while approximately 2/3 of the FPs are located in the protein–membrane interface adjacent to the true positives or on adjacent loops, possibly being TP predictions in reality.

For each one of the four test set proteins, we calculated the precision score independently, before and after considering that the FPs are possibly TPs due to their proximity to the membrane-interacting amino acids. We reclassified an FP amino acid as a TP if it was located within 3 Å distance from any atom of the membrane-interacting amino acids. The 3 Å radius was chosen because it is the distance of the interacting intermolecular forces and by expanding to this value, we would include amino acids that are directly interacting to the membrane-interacting amino acids. The resulting precision scores were significantly higher, with an improvement reaching 100% precision score in a few use cases (Supplementary Table S5). The extension to 3 Å is an empirical observation that improves the precision score, possibly because the limited experimental data hinder the definition of true positive labels of membrane-interacting amino acids (despite the generous definition of the IBS domain (Tubiana *et al.* 2022)). In the future, relabeling the dataset to extend the IBS regions by 3 Å around the current IBS regions could potentially enhance the accuracy of the trained models.

Furthermore, we compared our MLP models with the DREAMM (Chatzigoulas and Cournia 2022a, 2022b), MODA (Kufareva *et al.* 2014), PPM3 (Lomize *et al.* 2012, 2022), and PMIpred (van Hilten *et al.* 2024) tools. This comparison was conducted on the same four proteins in the test set, and we generated visualizations, additionally reporting the prediction times of all models (Supplementary Fig. S7 and Supplementary Table S6). Supplementary Table S7 illustrates the performance of all models for the four proteins collectively, from which we conclude that our MLP models exhibit superior performance with the model trained on ProtTrans embeddings displaying a 0.66 $F_{1}$ score and 0.64 MCC.

### 3.3 Model limitations using UniProt sequences

**Figure 4.**
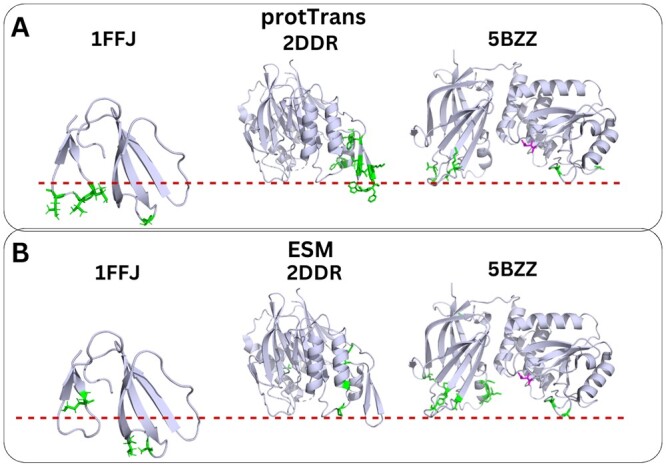
Predictions from the two MLP models trained on (A) ProtTrans embeddings and (B) ESM embeddings, for three proteins of the extra test set, with experimentally known protein–membrane interface regions. With green, we denote the predicted membrane-interacting amino acids. The membrane is depicted with a red–dotted line according to Nguyen *et al.* (2014), Ago *et al.* (2006), Dubovskii *et al.* (2001).

Next, we evaluated the performance of our models in the 33 proteins of the extra test set, for which we do not have the exact membrane-interacting amino acids, but only the region(s) that interact with the membrane have been verified experimentally or a few membrane-penetrating amino acids are known. For ∼1/3 of the proteins in this set, our MLP models correctly predicted the protein-membrane interface (Fig. 4, Supplementary Fig. S8A, and Supplementary Table S8). However, for the rest of the proteins, our models misclassified or did not predict any amino acid as membrane-interacting. All proteins of the extra test set (except for one that belongs to the PePrMInt dataset) belong to superfamilies to which our models were not trained on. These results demonstrate that the information in the dataset (nine protein superfamilies) is possibly inadequate to create a robust model and that our models lack generalization.

Finally, the MLP models were also assessed for the prediction of the protein–membrane interfaces of nine transmembrane enzymes (UniProt IDs: Q55487, P08842, Q99T05, P02919, P13516, O00767, Q03529, B9KDD4, O29867), but without success, as they cannot predict any membrane-interacting amino acid. In summary, the two MLP models are mostly limited to the specific protein superfamilies that they have been trained in, lacking generalization.

### 3.4 Predicting protein–membrane interfaces using sequences that are structurally resolved

Next, we extended our investigation to predict protein–membrane interfaces using sequences that are structurally resolved and reported in the PDB. These sequences represent a different source of protein structure data compared to the UniProt sequences used in our previous experiments and are often shorter due to containing only folded domains. We split the dataset as reported in Section 2.2 and trained the LGBM classifier—which was one of the most promising algorithms in the UniProt datasets in terms of training time (3× faster than MLP), configuration, and performance (<0.05 difference in $F_{1}\;$score compared to MLP, Tables 1 and 2)—to predict protein–membrane interfaces using PDB sequences in both datasets (ProtTrans & ESM). Detailed information on the search space and optimal hyperparameters on each dataset can be found in Supplementary Table S9 in the Supplementary material. The model trained on ProtTrans embeddings achieved an $F_{1}$ score of 0.8 and an MCC of 0.77, while the model trained on ESM embeddings attained an $F_{1}$ score of 0.77 and an MCC of 0.74.

**Figure 5.**
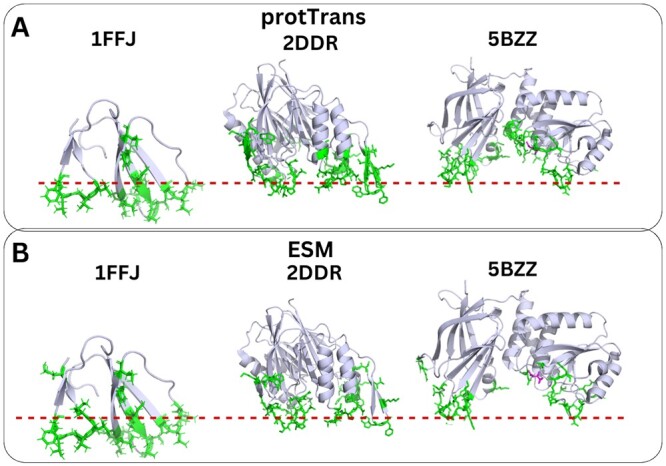
Predictions from the two LGBM models trained on (A) ProtTrans embeddings and (B) ESM embeddings in the dataset containing PDB sequences, for three proteins of the extra test set, with experimentally known protein–membrane interface regions. With green, we denote the predicted membrane-interacting amino acids. The membrane is depicted with a red–dotted line according to Nguyen *et al.* (2014), Ago *et al.* (2006), Dubovskii *et al.* (2001).

To further assess the predictive capabilities of our models, we visualized the prediction results on proteins of the extra test set (that includes 32 proteins from families not present in the training set), in which the putative protein–membrane interface has been proposed in the literature (Fig. 5 and Supplementary Fig. S8B). Intriguingly, we observed that the models trained on PDB sequences are more robust to unknown PMP superfamilies compared to the models trained on UniProt sequences – compare Fig. 4, Fig. 5, and Supplementary Fig. S8A and Fig. S8B. Moreover, we observed that the models trained on ESM and ProtTrans embeddings predicted similar membrane-interacting amino acids in the visualizations of the test set and extra test set proteins (compare Fig. 5A with Fig. 5B), with the model trained on ProtTrans embeddings predicting more amino acids as membrane-interacting. Both models had difficulty in predicting membrane-interacting amino acids in proteins <35 amino acids (i.e. PDB IDs: 1LA4, 1S6X, 2MH1). In summary, by utilizing PDB sequences, we demonstrate an improvement in the prediction of membrane-interacting amino acids and the development of more robust models.

## 4 Discussion and conclusions

Characterizing interactions at the protein–membrane interface is crucial, as abnormal peripheral protein–membrane attachment is involved in the onset of many diseases. Previously, we trained an ensemble ML classifier model (DREAMM) (Chatzigoulas and Cournia 2022a, 2022b) that predicts membrane-penetrating amino acids with high accuracy, which however, may require large computational resources and time, depending on the protein size. Therefore, in this paper, we test whether pLMs can produce results of similar accuracy for predicting membrane–interacting amino acids compared to the current state-of-the-art methods, but with fewer resources [such as ESMFold (Lin *et al.* 2023) compared to AlphaFold (Jumper *et al.* 2021)]. One caveat is that training an ML model using pLM embeddings to predict membrane–interacting amino acids needs big data in the training set, which however, proved to be scarce in the case of experimental protein–membrane interactions for PMPs.

In this study, we initially trained our models using sequences from UniProt and annotated them based on PDB structures. Using available experimental data from the PePrMInt and DREAMM datasets, we tested the predictive ability of pLMs to identify membrane–interacting amino acids. Specifically, using pLM embeddings as features, we trained MLP models that demonstrated excellent speed and accuracy in predicting membrane-interacting amino acids, albeit only for the nine protein superfamilies represented in the training set. A comparison of our MLP models with the DREAMM tool verified that our models exhibit swift outcomes without depending on the sequence length (Supplementary Table S6). For four randomly chosen proteins of the test set, that we used for visualization and comparison to the other state-of-the-art tools, our MLP models displayed increased accuracy in predicting the membrane-interacting amino acids. However, our models predicted correctly the protein–membrane interfaces in only ∼1/3 of the PMPs that belong to PMP superfamilies that do not exist in the training set, indicating that deep learning models face challenges to predict a property if it is not in the training set. Although our MLP models are protein superfamily-specific due to our dataset, they successfully predicted sporadic membrane-interacting amino acids in several protein superfamilies that were not included in the training set. This suggests that the models are not overfitted, but they are not robust enough to predict complete protein–membrane interfaces of unknown protein superfamilies.

To address the impact of including only structurally-resolved protein sequences on predictive performance, we subsequently created a dataset using PDB sequences. This new dataset aimed to mitigate the issue associated with unresolved protein regions, because the uncrystallized sequence regions were labeled as non-membrane-interacting, possibly misinforming our models, since these regions might contain membrane-interacting amino acids. The robustness and performance of the LGBM models increased, indicating a decreased dependence on superfamily-specific datasets, as evidenced by visualizations of proteins from unknown superfamilies with experimentally known membrane–interacting regions. The improved results of this methodology emphasized the importance of dataset selection and the information it contains. However, while the models trained on PDB sequences are more robust to unseen PMP superfamilies compared to models trained on UniProt sequences, they are still not as robust as DREAMM or other physics-based calculations. Nevertheless, this is the first proof of concept study, which shows that pLMs can be used for the prediction of membrane-interacting amino acids in PMPs, provided abundant experimental protein–membrane interfaces data.

Given the outcomes of our current study, some directions could be further explored to enhance the performance and generalization of the MLP models. One promising direction is to fine-tune the pre-trained language models on the PePrMint dataset (Tubiana *et al.* 2022). Fine-tuning allows to adapt the knowledge and representations learned by large-scale pLMs to a specific domain, potentially capturing more complicated patterns and improving the models' predictive capabilities. By fine-tuning pLMs on PePrMint dataset, one could potentially tailor the models to better utilize the unique characteristics of membrane–interacting amino acids. This process could potentially mitigate the limitations of relying solely on a small number of superfamilies and lead to improved predictions for protein superfamilies not included in the original dataset.

Moreover, the performance of the ML classifier models is highly reliant on the features/information utilized in this study. pLMs generate embeddings that contain encoded functional and structural information of protein sequences, which is crucial in the decision-making process of the ML models. In the current task, features relevant to the physicochemical properties of the amino acids are potentially more suitable, such as hydrophobicity, solvent exposure surface area, secondary structure element, etc. (Chatzigoulas and Cournia 2022b). Therefore, a future direction worth exploring is combining pLM embeddings with the physicochemical properties of amino acids. This combination of features could result in a more complete description of protein-membrane interfaces that may enhance the performance of the ML models.

Close inspection of the results revealed that many of the false positive predictions are possibly true positives as they are adjacent to the membrane-interacting amino acids. Using a cutoff radius of 3 Å around the membrane-interacting amino acids to reclassify the neighboring FP amino acids as TP elevated significantly the precision scores. Although the IBS domain is already extended beyond the experimentally-determined membrane-interacting amino acids, the limited experimental data and the absence of true positive labels for membrane-interacting amino acids provide the possibility of an even further extension of the IBS. From a future perspective, relabeling the dataset to increase the IBS regions by 3 Å around the existing IBS could possibly result in training models with increased accuracy.

Overall, the study has shed light on the challenges associated with studying and understanding protein–membrane interactions, particularly due to the limited knowledge of membrane-binding domains in PMPs. To address these challenges and improve the accuracy and prediction time for protein–membrane interface analysis, the application of artificial intelligence techniques within the context of natural language processing (NLP) has emerged as a promising avenue. In this study, we described an ML methodology for predicting membrane-interacting amino acids using pLMs. The evaluation of the two MLP models trained on ESM and ProtTrans embeddings for the datasets with UniProt sequences, yielded an $F_{1}$ score = 0.68 with and an MCC = 0.65 for the ProtTrans feature embeddings, and an $F_{1}$ score = 0.58 with and an MCC = 0.56 for the ESM feature embeddings. Training two LGBM models on ESM and ProtTrans embeddings for the datasets with PDB sequences, yielded an $F_{1}\;$score = 0.8 and an MCC = 0.76 for the ProtTrans embeddings, and an $F_{1}$ score = 0.77 and an MCC = 0.74 for the ESM embeddings, for correctly predicting the membrane-interacting amino acids.

To conclude, the results indicate that artificial intelligence, specifically NLP techniques, can make a significant contribution in the research community to tasks related to protein–membrane interaction, resulting in a substantial reduction in computing time compared to traditional non-deep learning techniques. As available data increase and language models continue to improve and outperform traditional algorithms, their usage will likely rise in the future in multiple areas of biomedical interest (Brown *et al.* 2020, Al‐twairesh 2021, Kalyan *et al.* 2022).

## Supplementary Material

vbae078_Supplementary_Data

## References

[vbae078-B1] Ago H , Oda M, Takahashi M et al Structural basis of the sphingomyelin phosphodiesterase activity in neutral sphingomyelinase from *Bacillus cereus*. J Biol Chem 2006;281:16157–67. 16595670 10.1074/jbc.M601089200

[vbae078-B2] Akiba T , Sano S, Yanase T et al Optuna: a next-generation hyperparameter optimization framework. In: *Proceedings of the *25th* ACM SIGKDD International Conference on Knowledge Discovery Data Mining*, Anchorage, AK, USA. 2019.

[vbae078-B3] Al‐twairesh N. The evolution of language models applied to emotion analysis of Arabic tweets. Information 2021;12:84.

[vbae078-B4] Berman HM , Westbrook J, Feng Z et al The protein data bank. Nucleic Acids Res 2000;28:235–42. 10592235 10.1093/nar/28.1.235 PMC102472

[vbae078-B5] Boes DM , Godoy-Hernandez A, McMillan DGG et al Peripheral membrane proteins: promising therapeutic targets across domains of life. Membranes 2021;11:346. 34066904 10.3390/membranes11050346 PMC8151925

[vbae078-B6] Brown TB , Mann B, Ryder N et al Language models are few-shot learners. *Adv Neural Inf ProcessSyst* 2020;33.

[vbae078-B7] Chatzigoulas A , Cournia Z. DREAMM: a web-based server for drugging protein–membrane interfaces as a novel workflow for targeted drug design. Bioinform2022a;38:5449–51. 10.1093/bioinformatics/btac680 PMC9750117 36355565

[vbae078-B8] Chatzigoulas A , Cournia Z. Predicting protein–membrane interfaces of peripheral membrane proteins using ensemble machine learning. Brief Bioinform2022b;23:bbab518. 35152294 10.1093/bib/bbab518 PMC8921665

[vbae078-B9] Chen T , Guestrin C. XGBoost: a scalable tree boosting system. In: *Proceedings of the 22nd acm sigkdd International Conference on Knowledge Discovery and Data Mining*, San Francisco, California, USA. 2016.

[vbae078-B10] Chollet F. Keras. 2015. https://keras.io (10 January 2024, date last accessed).

[vbae078-B11] Cox AD , Fesik SW, Kimmelman AC et al Drugging the undruggable RAS: mission possible? Nat Rev Drug Discov 2014;13:828–51. 25323927 10.1038/nrd4389 PMC4355017

[vbae078-B12] DeLano WL. *The PyMOL Molecular Graphics System*. Version 2.3. Schrödinger LLC, 2020.

[vbae078-B13] Dubovskii PV , Dementieva DV, Bocharov EV et al Membrane binding motif of the P-type cardiotoxin. J Mol Biol 2001;305:137–49. 11114253 10.1006/jmbi.2000.4283

[vbae078-B15] Elnaggar A , Heinzinger M, Dallago C et al ProtTrans: Toward understanding the language of life through self-supervised learning. IEEE Trans Pattern Anal Mach Intell 2022;44:7112–27. 34232869 10.1109/TPAMI.2021.3095381

[vbae078-B16] Fu L , Niu B, Zhu Z et al CD-HIT: accelerated for clustering the next-generation sequencing data. Bioinformatics 2012;28:3150–2. 23060610 10.1093/bioinformatics/bts565 PMC3516142

[vbae078-B17] Jumper J , Evans R, Pritzel A et al Highly accurate protein structure prediction with AlphaFold. Nature 2021;596:583–9. 34265844 10.1038/s41586-021-03819-2 PMC8371605

[vbae078-B18] Kalyan KS , Rajasekharan A, Sangeetha S. AMMU: a survey of transformer-based biomedical pretrained language models. J Biomed Inform 2022;126:103982. 34974190 10.1016/j.jbi.2021.103982

[vbae078-B19] Ke G , Ming Q, Finley T et al LightGBM: a highly efficient gradient boosting decision tree. *Adv Neural Inf Process Syst* 2017;30.

[vbae078-B20] Knight JS , Mazza LF, Yalavarthi S et al Ectonucleotidase-mediated suppression of lupus autoimmunity and vascular dysfunction. Front Immunol 2018;9:1322. 29942314 10.3389/fimmu.2018.01322 PMC6004379

[vbae078-B21] Kufareva I , Lenoir M, Dancea F et al Discovery of novel membrane binding structures and functions. Biochem Cell Biol 2014;92:555–63. 25394204 10.1139/bcb-2014-0074 PMC4267288

[vbae078-B22] Lemaître G , Nogueira F, Aridas CK et al Imbalanced-learn: a python toolbox to tackle the curse of imbalanced datasets in machine learning. J Mach Learn Res 2017;18:1–5.

[vbae078-B23] Lin Z , Akin H, Rao R et al Evolutionary-scale prediction of atomic level protein structure with a language model. Science 2023;379:1123–30. 36927031 10.1126/science.ade2574

[vbae078-B24] Lomize AL , Todd SC, Pogozheva ID. Spatial arrangement of proteins in planar and curved membranes by PPM 3.0. Protein Sci 2022;31:209–20. 34716622 10.1002/pro.4219 PMC8740824

[vbae078-B25] Lomize MA , Pogozheva ID, Joo H et al OPM database and PPM web server: resources for positioning of proteins in membranes. Nucleic Acids Res 2012;40:D370–6. 21890895 10.1093/nar/gkr703 PMC3245162

[vbae078-B26] Milella M , Falcone I, Conciatori F et al PTEN: multiple functions in human malignant tumors. Front Oncol 2015;5:24. 25763354 10.3389/fonc.2015.00024 PMC4329810

[vbae078-B27] Monje-Galvan V , Klauda JB. Peripheral membrane proteins: tying the knot between experiment and computation. Biochim Biophys Acta—Biomembr 2016;1858:1584–93. 10.1016/j.bbamem.2016.02.018 26903211

[vbae078-B28] Nguyen HN , Afkari Y, Senoo H et al Mechanism of human PTEN localization revealed by heterologous expression in *Dictyostelium*. Oncogene 2014;33:5688–96. 24292679 10.1038/onc.2013.507 PMC4041858

[vbae078-B29] PePrMInt dataset. 2022. https://github.com/reuter-group/tubiana_etal_2022 (5 February 2024, date last accessed).

[vbae078-B30] Rao R , Bhattacharya N, Thomas N et al Evaluating protein transfer learning with TAPE. Adv Neural Inf Process Syst 2019;32:9689–701. 33390682 PMC7774645

[vbae078-B31] Rao R , Liu J, Verkuil R et al MSA transformer. In: *Proceedings 38th International Conference on Machine Learning*, Vol. 139, pp. 8844–56. PMLR, 2021.

[vbae078-B32] Rao R , Meier J, Sercu T et al Transformer protein language models are unsupervised structure learners. *bioRxiv*, 2020. 10.1101/2020.12.15.42276.

[vbae078-B33] RCSB PDB - 2K5G. Solution NMR structure of protein encoded by gene BPP1335 from *Bordetella parapertussis*: Northeast Structural Genomics Target BpR195. 2008. https://www.rcsb.org/structure/2k5g (5 February 2024, date last accessed).

[vbae078-B34] Roderick SL , Chan WW, Agate DS et al Structure of human phosphatidylcholine transfer protein in complex with its ligand. Nat Struct Biol 2002;9:507–11. 12055623 10.1038/nsb812

[vbae078-B35] Snoek J , Larochelle H, Adams RP. Practical Bayesian optimization of machine learning algorithms. *Adv Neural Inf Process Syst* 2012.

[vbae078-B36] Steinegger M , Mirdita M, Söding J et al Protein-level assembly increases protein sequence recovery from metagenomic samples manyfold. Nat Methods 2019;16:603–6. 31235882 10.1038/s41592-019-0437-4

[vbae078-B37] Suzek BE , Wang Y, Huang H et al UniRef clusters: a comprehensive and scalable alternative for improving sequence similarity searches. Bioinformatics 2015;31:926–32. 25398609 10.1093/bioinformatics/btu739 PMC4375400

[vbae078-B38] Thorsell A-G , Lee WH, Persson C et al Comparative structural analysis of lipid binding START domains. PLoS One 2011;6:e19521. 21738568 10.1371/journal.pone.0019521 PMC3127847

[vbae078-B39] Tubiana T , Sillitoe I, Orengo C et al Dissecting peripheral protein–membrane interfaces. PLoS Comput Biol 2022;18:e1010346. 36516231 10.1371/journal.pcbi.1010346 PMC9797079

[vbae078-B40] UniProt Consortium. UniProt: a worldwide hub of protein knowledge. Nucleic Acids Res 2019;47:D506–15. 30395287 10.1093/nar/gky1049 PMC6323992

[vbae078-B41] Vanhaesebroeck B , Burke JE, Madsen RR et al Precision targeting of mutant PI3Kα in cancer by selective degradation. Cancer Discov 2022;12:20–2. 35022207 10.1158/2159-8290.CD-21-1411 PMC7612218

[vbae078-B42] van Hilten N , Verwei N, Methorst J et al PMIpred: a physics-informed web server for quantitative protein–membrane interaction prediction. *Bioinformatics* 2024;40:btae069. 10.1093/bioinformatics/btae069 PMC11212490 38317055

[vbae078-B43] Vaswani A , Shazeer N, Parmar N et al Attention is all you need. In: *31st Conference on Neural Information Processing Systems (NIPS 2017)*, Long Beach, CA, USA. 2017.

[vbae078-B44] White SP , Scott DL, Otwinowski Z et al Crystal structure of cobra–venom phospholipase a 2 in a complex with a transition-state analogue. Science 1990;250:1560–3. 2274787 10.1126/science.2274787

[vbae078-B45] Whited AM , Johs A. The interactions of peripheral membrane proteins with biological membranes. Chem Phys Lipids 2015;192:51–9. 26232665 10.1016/j.chemphyslip.2015.07.015

[vbae078-B46] Zukowska P , Kutryb-Zajac B, Toczek M et al The role of ecto-5′-nucleotidase in endothelial dysfunction and vascular pathologies. Pharmacol Rep 2015;67:675–81. 26321267 10.1016/j.pharep.2015.05.002

