# Supplementary material for: Using deep learning and large protein language models to predict protein–membrane interfaces of peripheral membrane proteins
Source: Bioinform Adv. 2024 May 28;4(1):vbae078. doi: 10.1093/bioadv/vbae078 (PMC11572487; doi:10.1093/bioadv/vbae078)
Supplement: vbae078_Supplementary_Data [file vbae078_supplementary_data.pdf]

Using deep learning and large protein language models to predict protein-membrane interfaces of peripheral membrane proteins

Dimitra Paranou[1,2], Alexios Chatzigoulas[1*], Zoe Cournia[1,2,*]

[1]Biomedical Research Foundation, Academy of Athens, 4 Soranou Ephessiou, 11527 Athens, Greece

[2]Department of Informatics and Telecommunications, National and Kapodistrian University of Athens, 15784 Athens, Greece

Protein alignment and annotation

Information in the datasets is provided with PDB codes and UniProt IDs and the chosen reference point in this project is the UniProt ID. Every UniProt ID corresponds to one or more PDB IDs, thus the class of each amino acid is related to the PDB sequence. To correctly transfer the information from the PDB sequence to the UniProt sequence, every corresponding PDB sequence was aligned with the UniProt sequence. The alignment procedure performs a local sequence alignment between two input protein sequences (UniProt and PDB sequences) using the Bio.pairwise2 module from Biopython library[1], the BLOSUM62 substitution matrix and specific gap penalties; a gap opening and a gap extension penalty is set to -10 and -1 respectively. Then for each aligned sequence, we annotate the corresponding amino acid based on the PDB sequence amino acid class. In case the UniProt sequence matches multiple PDB sequences, the union of the membrane-interacting amino acid class is taken. Figure S1 demonstrates an example with the aligned PDB sequences that correspond to the UniProt ID P14555, the class of each amino acid (IBS or not), and the final annotation on UniProt amino acids that emerged from the union of 1AYP and 1N28 membrane-interacting amino acid class.

Protein Language Models

A key feature of transformers is transfer learning, which is the application of knowledge gained from completing one task to help solve a different, but related, problem[2]. This, in combination with the fact that protein sequences are ideal for language models (LMs), has led many researchers to train new models to solve downstream tasks or to improve existing technologies[3–7]. Protein language models (pLMs) treat a protein sequence as a sentence and each amino acid as a single word, similar to Natural Language Processing (NLP).

Two of the most widely-known applications in the biology field are the ProtTrans[3] and evolutionary scale modeling (ESM)[6] models. ProtTrans successfully trained six NLP LMs (T5[8], Electra[9], BERT[10], Albert[11], Transformer-XL[12], and XLNet[13]) on protein sequences.

For ESM, researchers utilized large unsupervised LMs. They trained high-capacity transformer LMs on evolutionary data and observed that the model identified several key pieces of information such as homology, structural similarity, etc. It is worth noting, that ESMFold[14] achieved performance on par with that of AlphaFold[15] and RosettaFold[16], the two most known computational methods for predicting the 3D structure of proteins, while at the same time achieving a 600-fold speedup for results only marginally less accurate than those of AlphaFold2.

These models can be used for several tasks like secondary structure prediction, discovery of biological variations, capturing of biophysical features of amino acids, prediction of protein subcellular localization, and others.

Machine learning evaluation metrics

To choose the most appropriate model for each problem, evaluation metrics must be used to assess the performance of different models. Depending on the task and the kind of data being examined, machine learning can employ a wide range of different assessment criteria. For example, in regression tasks, the root mean square error is used to evaluate the model's effectiveness. In our case, we are dealing with a binary classification problem where we need to determine whether an amino acid interacts with the membrane or not. However, our dataset is imbalanced since a protein sequence contains numerous amino acids, but only a small fraction of them can interact with the membrane. Consequently, to evaluate the performance of such problems, specialized and advanced metrics are commonly used[17,18].

To measure the predictions with count values for each class, the confusion matrix can be used to assess where the model's errors were made (Figure S4). Having constructed the confusion matrix, several metrics can be calculated to quantify the performance of the model. The most common are the precision (Eq. 1) and the recall (Eq. 2) which compute what percentage of the predicted positive samples is truly positive and how good the model is at predicting the positive class, respectively.

$$precision = \frac{TP}{TP+FP} \qquad (1)$$

$$recall = \frac{TP}{TP+FN} \qquad (2)$$

The combination of recall and precision is called the F-score and is commonly used in tasks with imbalanced data. F_β is a general score (Eq. 3) that uses a positive real factor β, where β is chosen such that recall is considered β times as important as precision. When recall and precision are given equal weight, the resulting score is the F_1 score which is the harmonic mean of recall and precision (Eq. 4). F_1 score ranges from 0 to 1.

$$F_\beta = (1+\beta^2)\frac{precision*recall}{(\beta^2*precision)+recall} \qquad (3)$$

$$F_1 = 2*\frac{precision*recall}{precision+recall} \qquad (4)$$

Another metric is the Matthews correlation coefficient (MCC) (Eq. 5). The MCC metric ranges between -1 and 1 and is formulated as:

$$MCC = \frac{TP*TN-FP*FN}{\sqrt{(TP+FP)(TP+FN)(TN+FP)(TN+FN)}} \qquad (5)$$

Bayesian optimization

Bayesian optimization is a technique that seeks to find an optimal set of hyperparameters by balancing exploration and exploitation using a probabilistic model based on Bayesian statistics (Eq. 6). It aims to identify the global minimum of an objective function, which represents the performance of the model on a specific task, by developing a probabilistic model of the objective function based on the observed values of the hyperparameters. Compared to grid search or random search, Bayesian optimization can be more effective (Figure S4), especially for complex models with multiple hyperparameters. This is because it efficiently prioritizes the search for hyperparameters

likely to improve performance while also exploring other hyperparameters. By striking a balance between exploration and exploitation, Bayesian optimization helps identify the global minimum of the objective function[19].

$$\boldsymbol{P(A|B) = \frac{P(B|A) * P(A)}{P(B)}} \qquad (6)$$

Supplementary Figures

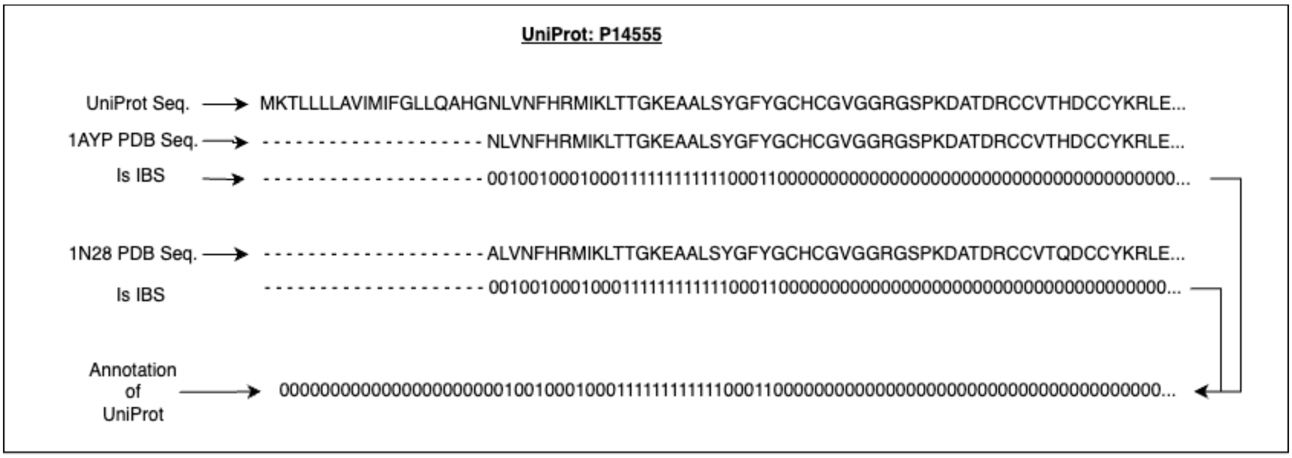

Figure S1: A UniProt sequence with its corresponding PDB sequences after alignment and their relative membrane-interacting amino acids (IBS or not). All the membrane-interacting classes are merged and the UniProt sequence is annotated.

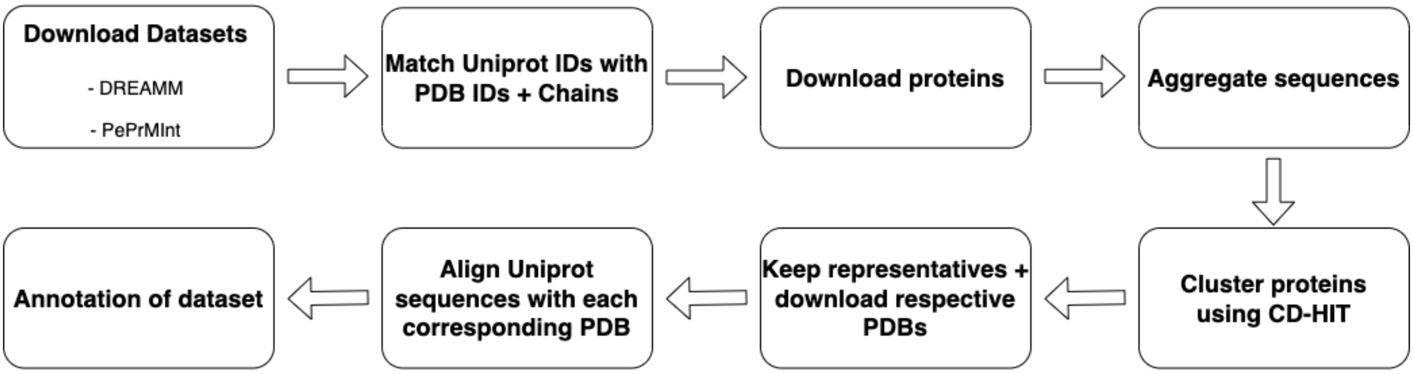

Figure S2: Pipeline for the dataset preparation containing UniProt sequences.

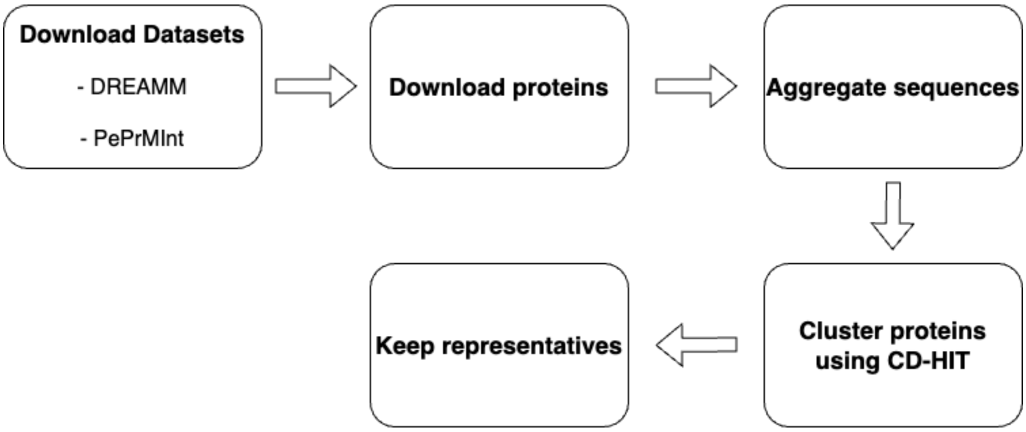

Figure S3: Pipeline for the dataset preparation containing the Uniprot ID sequence part that is resolved in the respective PDB entries. The existing annotation of the PePrMInt and DREAMM membrane-binding sites was used.

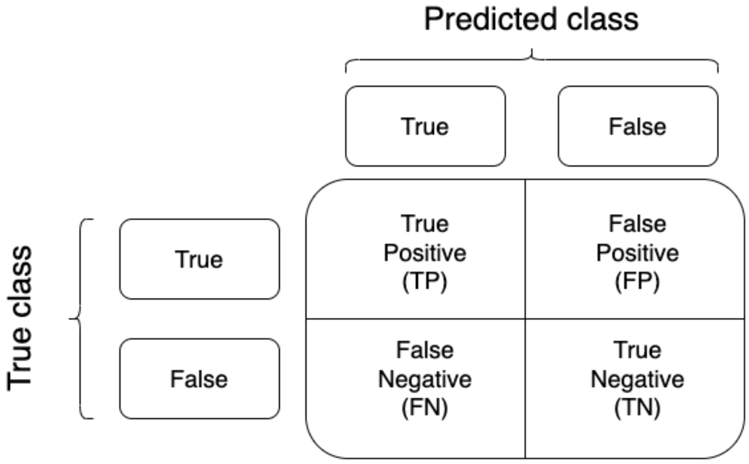

Figure S4: The Confusion matrix. The rows correspond to the actual labels and columns to the predicted classes.

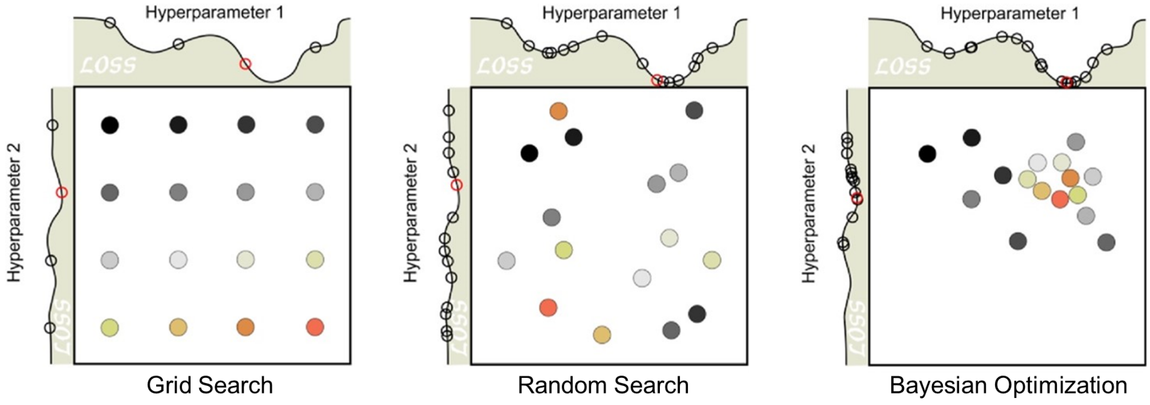

Figure S5: Hyperparameter optimization of two parameters with sixteen search trials in the three different search methods[20]. The bullets represent the set of parameters that are tested in each case and the color indicates the number of trials (1st trial is the black bullet – 16th trial is the orange bullet).

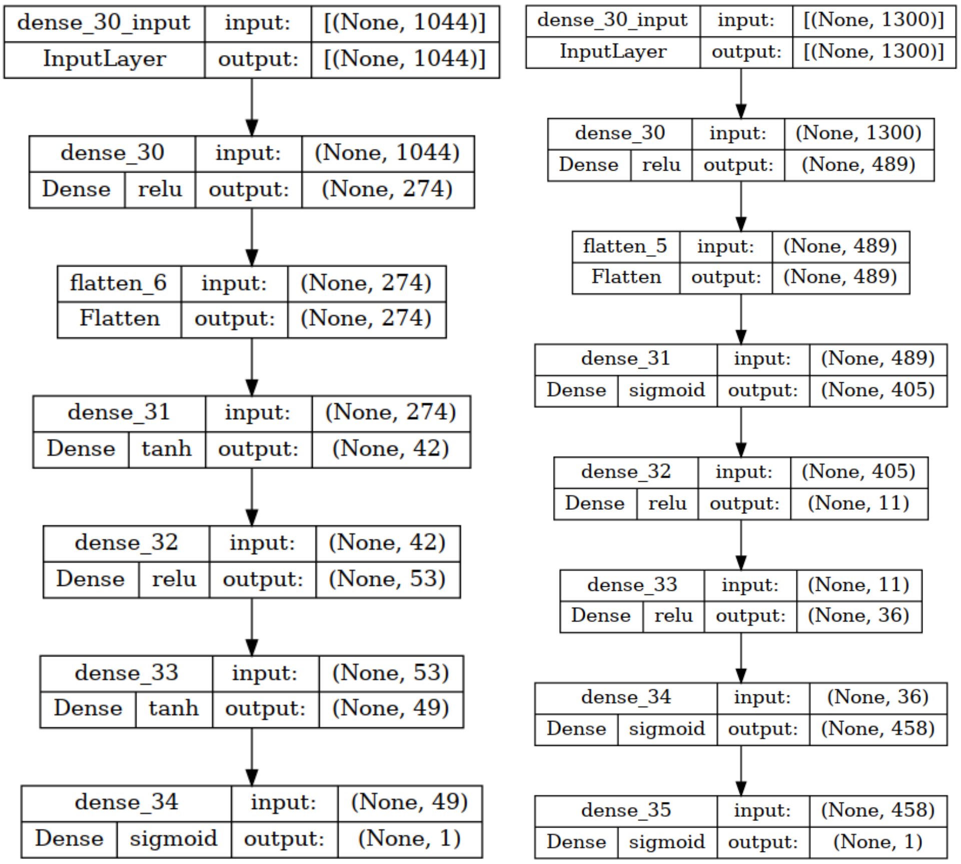

Figure S6: Architecture of MLP models that were constructed based on the best hyperparameters. On the left we show parameters of the ProtTrans model and on the right of the ESM model.

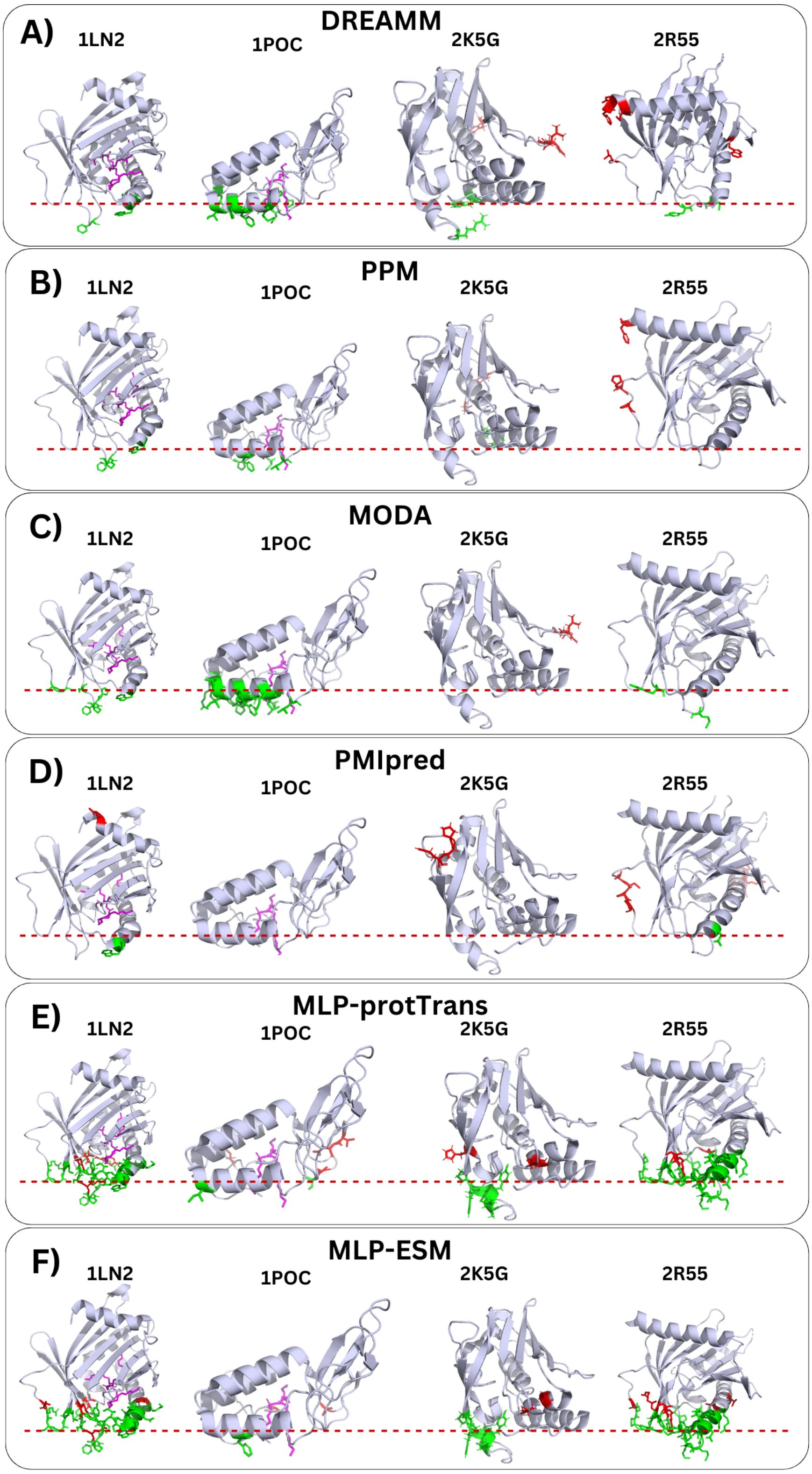

Figure S7: The predictions of four proteins of the test set from A) DREAMM, B) PPM3, C) MODA, D) PMIpred, and the MLP models trained on E) ProtTrans embeddings, and F) ESM embeddings. With green, we denote the TP, with red the FP, and with purple the ligands. The putative membrane plane is depicted as a red-dotted line and is aligned with the principal axis of all amino acids labeled as IBS in Tubiana et al[21].

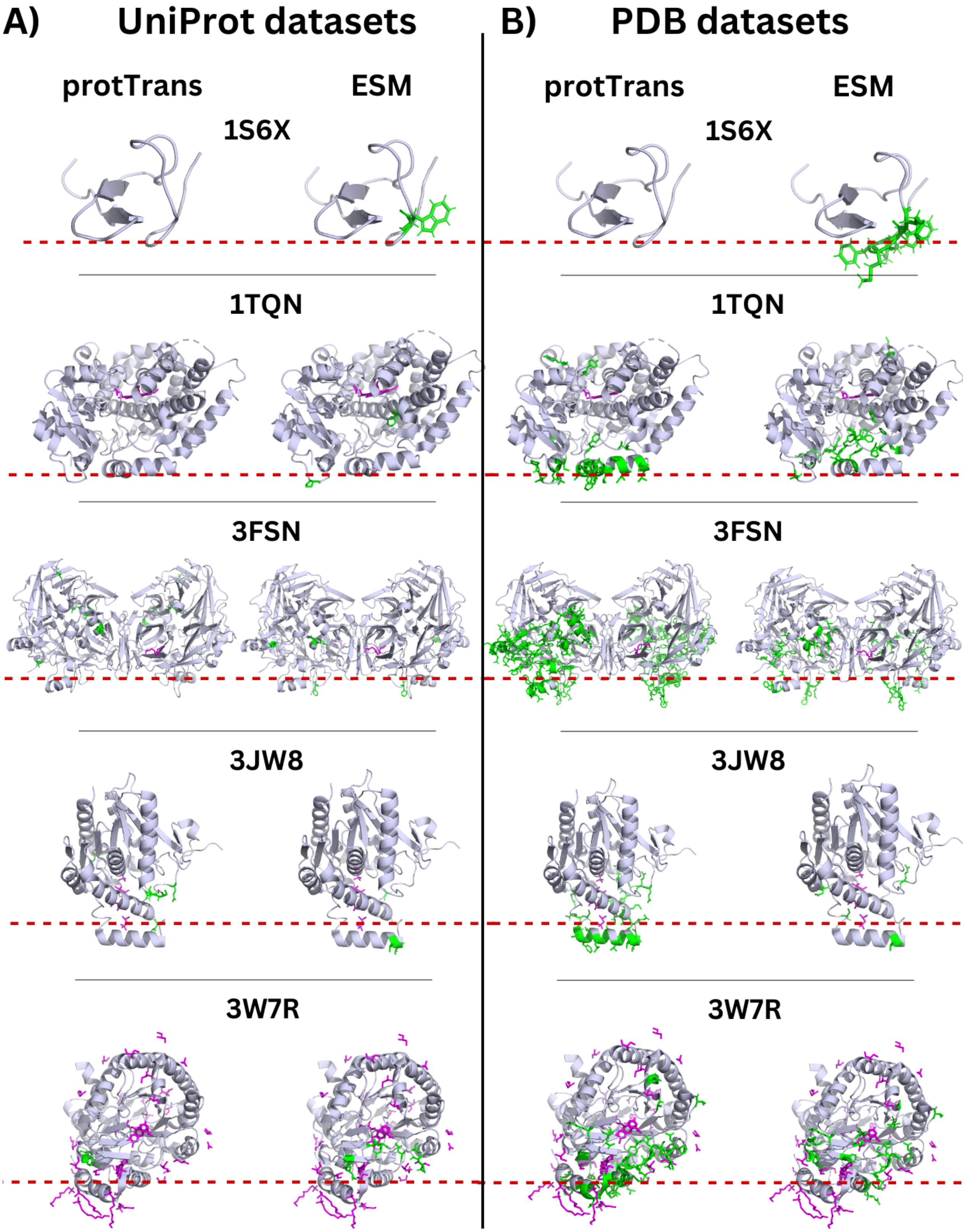

Figure S8: The predictions of five proteins of the extra test set from A) MLP trained on the UniProt datasets and B) LGBM trained on the PDB dataset (ProtTrans embeddings (left) and ESM embeddings (right), respectively). With green, we denote the predictions of the models, and with purple the ligands. The putative membrane plane is depicted as a red-dotted line according to Ref [22–26].

Supplementary Tables

Table S1: A part of the dataset with ProtTrans embeddings as features, and the one-hot encoding of the categorical amino acid feature. The final column is the class of each amino acid.

#	UniProt_id	Amino acid	Feature1	Feature2		Feature1024	A	C	K		L	Is_IBS
0	P14555	M	0.49	-1.54		1.32	0	0	0		0	0
1	P14555	K	1.22	0.89		-0.2	0	0	1		0	0
2	P14555	T	-0.34	1.02	…	0.05	0	0	0	…	0	0
3	P14555	L	0.89	-0.05		0.73	0	0	0		1	0
4	P14555	L	0.23	1.03		1.2	0	0	0		1	0
5	P14555	L	0.44	0.53		1.99	0	0	0		1	1

Table S2: The peripheral membrane protein Uniprot IDs of the training, validation, and test sets.

Set	Uniprot IDs
Training	O01761, Q9JKS6, Q60841, O75962, P21359, Q12802, P26039, V5M2P5, O15020, P00451, A0A0H2UP19, P12259, P97479, P32639, O75643, Q8XM24, O75923, Q9BZ29, D3ZJP6, Q9ERC5, Q3T552, A0A0H2YST8, Q5T5U3, D4QAP3, Q6ZPE2, Q14185, Q8DR60, Q04205, E5RWQ2, Q62768, Q15811, Q6ZPF3, Q8WZ64, Q61194, A0A0H2UNT5, Q8TCU6, P26831, Q9JIR4, P71140, Q13009, Q9JIS1, Q9NZN5, O15085, Q9Y2I1, Q6ZNL6, Q7SZN0, O75747, Q63HR2, Q69ZL1, Q62868, Q6EDY6, Q9ULU8, Q07889, P46934, Q96L93, Q68CZ1, P10686, Q96KN7, Q70E73, Q45712, P35568, Q01970, Q16760, P49796, Q99490, Q00722, P16480, P05068, Q08236, Q8XMY5, B3LEP7, Q64096, Q9QWY8, F2YQ19, Q9NQW6, Q0PRN1, Q9HAU0, Q8IX03, Q9Y3M8, P48736, P14090, Q9ZA17, Q8Y4J2, Q9Y2J2, O69230, P51584, P42337, Q54873, Q91VS8, Q8BTI9, P29323, Q00944, Q9Y2H5, Q9Y5B9, A0A0H2US34, P32558, Q56F26, Q82PP4, Q8VNN2, P00723, Q8A2X6, Q70SY0, Q8KRF6, Q9KG76, Q8IWE5, Q0TR53, Q6P4T1, Q7WTN6, Q8N960, Q92974, Q9VFS5, P94286, Q6DN90, Q93RE7, A3DHG6, Q9Y5W7, A0A075B5H6, O60462, Q59290, Q9BYX2, A0FGR8, Q08345, Q92888, P77847, Q96J02, P0C2S1, O94806, Q6ZUM4, Q9BZF1, Q6DN12, Q61097, P50570, P11171, Q8AAK6, Q16832, D1GCC6, P15498, A3DK57, Q840C0, Q8N4X5, A6KXE5, P10477, O95267, Q93IE7, Q8AB22, Q9H4M7, Q8K4I3, P22346, Q9HCE7, P10688, Q9BRR9, P47712, Q9BXB4, B3CET4, Q15027, P09216, O00522, Q99PV3, A4I5U9, Q8A916, Q92556, Q3J126, Q5JSP0, Q9UH99, O54924, Q8R5F8, Q08945, Q02111, Q5LJ68, P05129, P9WG63, Q5LIC7, Q9NR80, P21146, P40485, P47709, O52780, P0CS93, Q96AC1, Q9RIK9, P16278, P79134, Q96C24, Q8VRK8, Q13613, Q2PHL4, Q3ZC95, Q82L26, P08236, Q4AE70, P04049, Q02834, P42331, Q9ZB22, P32776, Q9WXN1, P45796, P0A377, P18887, Q9H0H5, Q9L5A4, Q9Y5P4, O07653, Q9JID9, Q9Y217, Q8WXI4, Q59675, Q9X0S8, P05804, P46662, A0A0H2UN19, Q9Y5X1, Q13322, Q6DN99, P40748, A2AR50, Q9UH65, Q51815, Q9NPI6, Q8WV41, Q9L9D7, G0SHK5, Q06696, H6WCZ0, A0A0H2WZL3, D8DVU6, O15530, P10820, P29366, P32780, Q96L92, Q00019, Q91X46, Q13596, Q9BPZ7, Q8WU20, Q18PE0, Q61234, Q9ERE3, P16559, Q4W8M3, Q8A5P6, G0L2L9, P97465, P31751, B7GNN8, Q9BSW7, Q15036, P52757, P21956, Q8AAM3, Q8A3J5, P40161, Q38CF2, Q14849, Q9BSQ5, Q8A9F0, Q7L8C5, Q9ERS5, Q9H2B2, P21579, Q5EBH1, O60496, B1H267, O43581, O43739, P14598, Q9H0F6, Q9UNH6, Q86VN1, E0RVY7, Q8NFA2, O75689, Q9W1H5, A7LSX5, C4QH88, D6MSV6, Q96MF2, B3PDE5, Q86WV1, B3PIB0, P09394, Q9NYT0, P08567, B3A043, P25335, Q5L9W9, P04272, Q15080, O95433, Q5LFR2, Q9SSK9, A0A0J9X278, P08954, P34024, Q9SYT0, A6LIT8, P32912, A0A6L7H2E6, P45723, Q5LX22, Q2LK81, Q8I914, D7RFJ9, Q2PA00, Q4VPP2, Q9P104, Q8C4Q6, Q8A2Z3, Q9HB20, Q80UW2, P17063, Q9ULZ2, P49675, Q9UN19, Q9CR95, A0A6N4SPL7, P53810, Q9Y5W9, Q9RZE3, A6L916, P42530, P53068, A9CLR1, A9CG82, Q5JGZ3, A0A0H2V2B5, Q9QYE9

Validation	Q8H1L1, Q1MFM4, Q9I4D2, G7J032, P52778, Q3J4M4, Q5HLI9, Q8NN40, O04298, A1U5H9, Q9XG81, Q2K6S8, Q2Y8N9, Q5QL47, P80966, Q6UV28, Q47KK8, Q9A7I7, Q5SK03, Q7NY36, Q9C8S6, Q9Y9R3, A0A6N4SU23, Q484T9, O31806, P14555, Q5LN61, Q9Y547, Q98IT8, Q689C4, Q81AY6, Q8DVN6, A0A6N4SXV3, A0KKT0, Q973T5, Q49US3, Q7CZ16, E2FYL5, Q64YT5, Q9P805, P0C0B0
Test	P04183, Q89ZG6, Q12517, Q53W25, Q99JV5, Q9PPP5, Q8VZS8, P59095, A1ZAW5, Q9UKL6, Q9NSY2, Q9P4F6, Q67A25, Q8PPZ5, Q9HJ63, O14713, Q96L94, B8LIX8, Q9UM13, A0A1C9V3S9, Q9WYN2, Q7WAN9, Q8KNE9, Q8KNF0, B9PKK4, Q82XK1, Q8PZJ2, Q9F6D3, A0A0H2XIZ7, A0A6N4SQ07, P00630, Q9ZLJ5, A1RA60, O15496, B9PJE6, P93330, Q08826, Q9UMY4, Q98FZ2, Q832L1, A1JSS7
Extra Test	P61914, Q15075, O16025, Q96QK1, Q960X8, P12530, P00735, P40343, O24592, P05979, O88339, P22637, Q28175, P08684, P0C2E9, Q9LCB2, P60484, P0C216, Q02127, P20932, P02749, P11889, Q77DJ6, P00803, Q99685, P49638, Q9NZD2, P00720, P12724, P12104, P56254, P60980, P01441

Table S3: The peripheral membrane protein PDB IDs of the training, validation, and test sets.

Set	PDB IDs
Training	2ROV, 3CIH, 2W91, 3NSJ, 2D9Z, 3QAR, 2VTF, 3PVL, 1MAI, 2ZXQ, 2E3M, 3GM8, 1ICX, 3CXL, 2D9W, 3H4X, 2COC, 1JI6, 1BAK, 3W5M, 4XRW, 1D7P, 5BRL, 2L8O, 1KMD, 1QQG, 2V14, 2XOM, 2DFK, 2YC2, 1WKY, 2LUL, 3L12, 4XUP, 2YRB, 3Q63, 4CU7, 1NKG, 2RGN, 2R83, 1KZG, 4IFS, 3HNM, 1UXZ, 2L9P, 2WAA, 3GGL, 3US7, 1K3I, 1FB8, 2YF0, 1X53, 2YUU, 4HHV, 1K42, 1XFS, 1PFJ, 1TBN, 1X05, 2LUZ, 2FJL, 2ADZ, 1GQP, 2XQX, 3IE5, 2RAJ, 3PU2, 2Y8J, 3TVV, 3W57, 4MGQ, 1W0N, 2DDU, 2I9Y, 1LE6, 4BFR, 4LPL, 4QPW, 3SEE, 3WQB, 1XPW, 2Y8K, 3OG2, 4A5K, 2QKM, 5D27, 4C0A, 4MDU, 3OH8, 3TVR, 2VZS, 3CNW, 1UY1, 2W5F, 2GCL, 4QAW, 4JX0, 2P0F, 2LOZ, 3III, 2BGO, 3PUT, 3AU5, 2KCZ, 2LYD, 4OYU, 2PSO, 2B3R, 4R7K, 5E4M, 3P9V, 3HW2, 3MBW, 4IAP, 4DXT, 4EVF, 3CH0, 1GUI, 3RWS, 3ELI, 1H6H, 1Q67, 4CVU, 3C7G, 1POC, 3FBK, 2LIO, 1U5E, 3P51, 4ARY, 1ZXF, 2WG7, 2K5G, 2WAB, 3NI8, 3F5R, 4ZXE, 2OQB, 2EP6, 2ENP, 2DHJ, 5F7U, 2ENJ, 4A42, 2LAK, 2O55, 4E8C, 2QPV, 2ZEW, 2W47, 1FOE, 2NS9, 3U37, 3JZY, 2I4K, 4KHA, 2QKG, 4HDQ, 3ZM8, 2E73, 1WFJ, 2CDP, 1V5P, 1ZC4, 1ULO, 1PLS, 4TXW, 2IL5, 4A6O, 2D4R, 1FAQ, 2OKX, 1WFM, 2LS6, 2M47, 2DMH, 4P2I, 2HTH, 4GN1, 2DTC, 2NN5, 3IQ2, 1H6X, 1X86, 3FO5, 2I1J, 1LN1, 4UI9, 1KQ6, 1XD4, 1CX1, 1SDD, 1FVZ, 4R7O, 1W1H, 2C26, 1V5U, 2Z0Q, 1V27, 3QSZ, 4F91, 3ODO, 4CHJ, 3UES, 1T17, 4GZU, 1FHO, 2J1F, 2ETT, 1W9S, 4EMO, 3OHM, 2M89, 2WWE, 1OH4, 2Q3X, 4WSF, 4KAX, 2LE1, 2LF2, 1CJY, 1RH8, 2R55, 4PF1, 1AOD, 3EYP, 4GWI, 2B8T, 1XX1, 4XVH, 4DX8, 2V6V, 1VU2, 3NNG, 2D4Q, 2ENQ, 3TFM, 3FN9, 1XUV, 1YDY, 1BHG, 4CHM, 1W7B, 3SKV, 4OEC, 2H7D, 3P0C, 2FFS, 3WNK, 1WGQ, 2W46, 2L73, 3RD6, 3L9B, 1J0W, 3KWU, 4B6D, 1LW3, 2AL6, 3Q6A, 5EFX, 2BK0, 1ZCC, 2JDA, 4LHS, 1WJM, 3NQH, 4D8M, 3BGA, 1M9I, 4BGD, 4CU9, 2CM6, 3I10, 1V61, 4JKK, 1XTE, 2MOU, 2DA0, 2CAY, 4WJ7, 2QQM, 1AOK, 1J84, 2HYX, 2PCS, 1UNP, 1Y8F, 2PQS, 4K2P, 3ULB, 2YRY, 1V5M, 3PUI, 3LUI, 2OTD, 4UAP, 3B7Y, 2ORW, 2LEQ, 4PZG, 3P0L, 3NO3, 2MFQ, 2LKO, 3W9K, 4XRT, 4AUP, 1GNY, 1V88, 1EUT, 3A21, 2CY4, 1Y5O, 2WZ8, 1TQZ, 3HPC, 2VQ5, 4K81, 2D9X, 4QAM, 4K17, 4CUB, 1VJH, 1KI1, 2WUH, 2GVI, 3D3A, 2W1U, 3ACF, 1BTK, 4ICX, 1DYN, 2LDK
Validation	1DK5, 2IWL, 1WI1, 1R79, 4AJW, 1DRO, 3THC, 1VD6, 2CJS, 2DLW, 2JE8, 1SG3, 2B79, 3CQO, 4LCV, 2COF, 3BJI, 2K2J, 3TFZ, 2RLO, 2COD, 3PFQ, 2HLE, 2VSZ, 1RFH, 4BGJ, 5EIY, 3ELO, 2KCJ, 1Z94, 3KDH, 3ML4, 3E2I, 1X1F, 5C70, 2QZ5, 1LNS, 1WG7, 2ROW, 3ABZ, 2HE7, 5FUY
Test	2Y7B, 3KS6, 1W99, 1PMJ, 4GXB, 4HAS, 2E26, 4TYZ, 3V1H, 3QVQ, 2W94, 3MZ2, 4NSW, 3P6B, 2FK9, 2Z0U, 4D0Q, 3L4C, 1I5P, 4LKS, 5I9J, 3MPX, 2NQ3, 3VOQ, 1NTY, 3WSO, 2DHI, 3PYC, 2V72, 2W9X, 3CMG, 2KEW, 3LJU, 2DHK, 4NPJ, 3FDW, 1KBE, 2II2, 2R55, 1LN1, 2K5G, 1POC
Extra Test	1COY, 2FNQ, 1S6X, 2DDR, 1FFJ, 1DVP, 1PFO, 3NPE, 3AKM, 3FSN, 3RZN, 1ES6, 1GYG, 2MH1, 1JOC, 1NL1, 1TQN, 3W7R, 2KS4, 3JW8, 1LA4, 2P0M, 1OIZ, 5HXW,3IIQ, 1H0A, 2AYL, 4X08, 1C1Z, 5F0P, 6BFG, 5BZZ

Table S4: The hyperparameters that were sampled for the five classifiers of this study for each dataset, the ranges that were searched in Optuna optimization, and the final best hyperparameters for each dataset.

Algorithm	Dataset	Hyperparameter ranges	Best hyperparameters identified by hyperparameter optimization
XGBoost*	ProtTrans	Objective: binary:logistic,<br>1e-4 ≤ Learning rate ≤ 1e-1<br>Booster: [gbtree, dart]<br>0.7 ≤ Gamma ≤ 1, step = 0.1<br>1e-8 ≤ Lambda ≤ 1<br>1e-2 ≤ Reg alpha ≤ 10<br>0.2 ≤ Subsample ≤ 1<br>0.5 ≤ Colsample bytree ≤ 1<br>1 ≤ Max depth ≤ 31, step = 2<br>1 ≤ Min child weight ≤ 10<br>32 ≤ Scale pos weight ≤ 42	Learning rate:<br>0.04838262352995109<br>Booster: dart<br>Lambda: 0.003935832973774852<br>Reg alpha: 5.883577941007524<br>Reg lambda: 8.276188412936722<br>Subsample: 0.7092613064434476<br>Gamma: 0.7999999999999999<br>Colsample bytree:<br>0.73377885289854<br>Max depth: 11<br>Min child weight: 2<br>Scale pos weight:<br>39.25157481352274
	ESM		Learning rate:<br>0.037776862175690346<br>Booster: dart<br>Gamma: 1.0<br>Lambda: 9.486854501008372e-08<br>Reg alpha: 2.7045282341272148,<br>Reg lambda: 6.356108313972932<br>Subsample: 0.7717790611371791<br>Colsample bytree:<br>0.50111392851597<br>Max depth: 9<br>Min child weight: 10<br>Scale pos weight:<br>34.149578682619

LGBM*	ProtTrans	Objective: binary,<br>Boosting type: gbdt,<br>Metric: binary_logloss<br>1e-4 ≤ Learning rate ≤ 1e-1<br>1 ≤ Max depth ≤ 32, step = 2<br>20 ≤ Number of leaves ≤ 100<br>0.5 ≤ Feature Fraction ≤ 1<br>0.5 ≤ Bagging Fraction ≤ 1<br>1 ≤ Bagging Freq ≤ 10<br>0 ≤ Lambda 1 ≤ 1<br>0 ≤ Lambda 2 ≤ 1<br>100 ≤ Number of iterations ≤ 2000, step = 100<br>32 ≤ Scale pos weight ≤ 42	learning_rate: 0.013806052880760<br>max_depth: 31<br>scale_pos_weight: 34.68346480<br>num_leaves: 84<br>feature_fraction: 0.7271452869<br>bagging_fraction: 0.7552953927<br>bagging_freq: 7<br>lambda_l1: 0.35802780689923<br>lambda_l2: 0.809829964286<br>num_iterations: 2000
	ESM		learning_rate: 0.019830123385066<br>max_depth: 11<br>scale_pos_weight: 39.01430421<br>num_leaves: 25<br>feature_fraction: 0.7196445907<br>bagging_fraction: 0.5802674860<br>bagging_freq: 6<br>lambda_l1: 0.86296338248<br>lambda_l2: 0.8870113126431<br>num_iterations: 1900
Balanced Random Forest*	ProtTrans	50 ≤ Number of estimators ≤ 500<br>Criterion: [gini, entropy]<br>1 ≤ Max depth ≤ 31, step = 2<br>2 ≤ Min samples split ≤ 20<br>1 ≤ Min samples leaf ≤ 20<br>0.1 ≤ Max features ≤ 1	Number of estimators: 476<br>Criterion: gini<br>Max depth: 31<br>Min samples split: 9<br>Min samples leaf: 1<br>Max features: 0.1000274146856
	ESM		Number of estimators: 498<br>Criterion: gini<br>Max depth: 31<br>Min samples split: 2<br>Min samples leaf: 1<br>Max features: 0.1001512074230
Single Layer Perceptron**	ProtTrans	1 ≤ Hidden layer sizes ≤ 1000<br>Activation: [relu, tanh, sigmoid]<br>Optimizer: [adam, sgd]<br>1e-5 ≤ Learning rate ≤ 1e-1	Hidden layer sizes: 412<br>Activation: tanh<br>Optimizer: adam<br>Learning rate: 0.000404
	ESM		Hidden layer sizes: 776<br>Activation: relu<br>Optimizer: adam<br>Learning rate: 0.0009146523695

Multi-Layer Perceptron**	ProtTrans	1 ≤ Numder of layers ≤ 4<br>4 ≤ Hidden layer size ≤ 512<br>1e-10 ≤ Weight decay ≤ 1e-3<br>Activation: [relu, tanh, sigmoid]<br>Optimizer: [adam, sgd]<br>1e-5 ≤ Learning rate ≤ 1e-1<br>10 ≤ Epochs ≤ 30<br>128 ≤ Batch size ≤ 512	Number of layers: 3<br>Weight decay: 2.695517781e-08<br>Number of units layer Input: 274<br>Activation input: relu<br>Number of units layer 0: 42<br>Activation 0: tanh<br>Number of units layer: 53<br>Activation 1: relu<br>Number of units layer 2: 49<br>Activation 2: tanh<br>Optimizer: Adam<br>Learning rate: 0.0018389654970<br>Epochs: 14<br>Batch size: 283
	ESM		Number of layers: 4<br>Weight decay: 8.695617261789e-05<br>Number of units layer input: 489<br>Activation input: relu<br>Number of units layer 0: 405<br>Activation 0: sigmoid<br>Number of units layer 1: 11<br>Activation 1: relu<br>Number of units layer 2: 36<br>Activation 2: relu<br>Number of units layer 3: 458<br>Activation 3: sigmoid<br>optimizer: Adam<br>Learning rate: 0.0285374828993805<br>Epochs: 25<br>Batch size: 171

* 200 trials

** 50 trials

Table S5: The precision score of the four test set proteins before and after reclassifying from FP to TP the amino acids that are possibly TPs due to their proximity to the membrane-interacting amino acids.

	**protTrans**	**ESM**	**PDB ID**
Precision score	0.9	0.81	1LN2
Recalculated Precision score	0.97	1	
Precision score	0.25	0.33	1P0C
Recalculated Precision score	0.25	0.33	
Precision score	0.71	0.78	2KG5
Recalculated Precision score	1	0.78	
Precision score	0.97	0.83	2R55
Recalculated Precision score	1	0.97	

Table S6: The predicted membrane-interacting amino acid indexes from DREAMM, PPM3, MODA, PMIpred, and the MLP models trained on ProtTrans embeddings, and ESM embeddings, for four proteins of the test set. With * we denote the false positive predictions. For homomers chain A was kept and for NMR structures the first model. All tools were used with the default parameters. In PPM3 we included heteroatoms. In PMIPred a neutral membrane was chosen. Amino acid numbering is consistent with the PDB structure.

Model	PDB	TP	FP	FN	TN	True labels	Predicted	Time
DREAMM[27]	1LN2	3	0	26	185	83, 86, 104, 105, 106, 107, 108, 110, 111, 146, 147, 148, 149, 150, 181, 182, 183, 184, 185, 186, 187, 188, 189, 190, 191, 193, 194, 195, 198	107, 186, 190	5 min
	1POC	8	0	19	204	1, 2, 3, 4, 5, 6, 9, 10, 11, 14, 15, 22, 23, 24, 51, 52, 54, 55, 76, 77, 78, 79, 81, 82, 83, 85, 86, 89, 90, 91, 92	1, 2, 11, 24, 78, 81, 82, 86	1 min
	2K5G	2	3	35	151	67, 68, 71, 72, 73, 74, 75, 76, 77, 78, 79, 80, 81, 82, 83, 84, 85, 86, 143, 147, 148, 166, 167, 168, 169, 170, 171, 172, 173, 174, 175, 176, 177, 178, 179, 180, 181	8*, 15*, 16*, 78, 169	3 min

	2R55	2	5	27	198	81, 84, 104, 105, 106, 107, 108, 109, 110, 111, 112, 113, 145, 146, 147, 148, 149, 182, 183, 184, 185, 186, 187, 188, 189, 190, 191, 192, 195	2*, 3*, 5*, 40*, 108, 141*, 183	4 min
PPM3[28]	1LN2	3	0	26	185	83, 86, 104, 105, 106, 107, 108, 110, 111, 146, 147, 148, 149, 150, 181, 182, 183, 184, 185, 186, 187, 188, 189, 190, 191, 193, 194, 195, 198	107, 108, 186	33 sec
	1POC	4	0	23	204	1, 2, 3, 4, 5, 6, 9, 10, 11, 14, 15, 22, 23, 24, 51, 52, 54, 55, 76, 77, 78, 79, 81, 82, 83, 85, 86, 89, 90, 91, 92	1, 4, 82, 90	12 sec
	2K5G	2	2	35	152	67, 68, 71, 72, 73, 74, 75, 76, 77, 78, 79, 80, 81, 82, 83, 84, 85, 86, 143, 147, 148, 166, 167, 168, 169, 170, 171, 172, 173, 174, 175, 176, 177, 178, 179, 180, 181	8*, 164*, 167, 170	26 sec

	2R55	0	4	29	198	81, 84, 104, 105, 106, 107, 108, 109, 110, 111, 112, 113, 145, 146, 147, 148, 149, 182, 183, 184, 185, 186, 187, 188, 189, 190, 191, 192, 195	2*, 140*, 141*, 143*	23 sec
MODA[29]	1LN2	7	0	22	185	83, 86, 104, 105, 106, 107, 108, 110, 111, 146, 147, 148, 149, 150, 181, 182, 183, 184, 185, 186, 187, 188, 189, 190, 191, 193, 194, 195, 198	106, 107, 108, 110, 147, 148, 186	5 sec
	1POC	14	0	13	204	1, 2, 3, 4, 5, 6, 9, 10, 11, 14, 15, 22, 23, 24, 51, 52, 54, 55, 76, 77, 78, 79, 81, 82, 83, 85, 86, 89, 90, 91, 92	1, 2, 3, 4, 5, 23, 24, 77, 78, 81, 82, 85, 86, 90	5 sec
	2K5G	0	2	37	152	67, 68, 71, 72, 73, 74, 75, 76, 77, 78, 79, 80, 81, 82, 83, 84, 85, 86, 143, 147, 148, 166, 167, 168, 169, 170, 171, 172, 173, 174, 175, 176, 177, 178, 179, 180, 181	15*, 16*	5 sec

	2R55	2	0	27	202	81, 84, 104, 105, 106, 107, 108, 109, 110, 111, 112, 113, 145, 146, 147, 148, 149, 182, 183, 184, 185, 186, 187, 188, 189, 190, 191, 192, 195	108, 183	7 sec
PMIPred[30]	1LN2	2	1	27	184	83, 86, 104, 105, 106, 107, 108, 110, 111, 146, 147, 148, 149, 150, 181, 182, 183, 184, 185, 186, 187, 188, 189, 190, 191, 193, 194, 195, 198	11*, 185, 186	3 sec
	1POC	0	0	27	204	1, 2, 3, 4, 5, 6, 9, 10, 11, 14, 15, 22, 23, 24, 51, 52, 54, 55, 76, 77, 78, 79, 81, 82, 83, 85, 86, 89, 90, 91, 92	-	3 sec
	2K5G	0	3	37	151	67, 68, 71, 72, 73, 74, 75, 76, 77, 78, 79, 80, 81, 82, 83, 84, 85, 86, 143, 147, 148, 166, 167, 168, 169, 170, 171, 172, 173, 174, 175, 176, 177, 178, 179, 180, 181	57*, 58*, 59*	14 sec

	2R55	1	9	28	193	81, 84, 104, 105, 106, 107, 108, 109, 110, 111, 112, 113, 145, 146, 147, 148, 149, 182, 183, 184, 185, 186, 187, 188, 189, 190, 191, 192, 195	141*, 142*, 143*, 144*, 187, 209*, 210*, 211*, 212*, 213*	4 sec
MLP ProtTrans	1LN2	26	3	3	182	83, 86, 104, 105, 106, 107, 108, 110, 111, 146, 147, 148, 149, 150, 181, 182, 183, 184, 185, 186, 187, 188, 189, 190, 191, 193, 194, 195, 198	49*, 83, 86, 104, 105, 106, 107, 108, 109*, 110, 111, 112*, 146, 147, 148, 149, 150, 181, 182, 183, 184, 185, 186, 187, 188, 190, 191, 193, 194	15 sec
	1POC	1	3	26	201	1, 2, 3, 4, 5, 6, 9, 10, 11, 14, 15, 22, 23, 24, 51, 52, 54, 55, 76, 77, 78, 79, 81, 82, 83, 85, 86, 89, 90, 91, 92	79, 104*, 109*, 122*	15 sec
	2K5G	10	4	27	150	67, 68, 71, 72, 73, 74, 75, 76, 77, 78, 79, 80, 81, 82, 83, 84, 85, 86, 143, 147, 148, 166, 167, 168, 169, 170, 171, 172, 173, 174, 175, 176, 177, 178, 179, 180, 181	64*, 69*, 70*, 72, 73, 74, 75, 80, 81, 82, 83, 84, 86, 144*	15 sec

	2R55	28	1	1	201	81, 84, 104, 105, 106, 107, 108, 109, 110, 111, 112, 113, 145, 146, 147, 148, 149, 182, 183, 184, 185, 186, 187, 188, 189, 190, 191, 192, 195	81, 84, 104, 105, 106, 107, 108, 109, 110, 111, 112, 113, 114*, 145, 146, 147, 148, 149, 182, 183, 184, 185, 186, 187, 188, 189, 191, 192, 195	15 sec
MLP ESM	1LN2	26	6	3	179	83, 86, 104, 105, 106, 107, 108, 110, 111, 146, 147, 148, 149, 150, 181, 182, 183, 184, 185, 186, 187, 188, 189, 190, 191, 193, 194, 195, 198	86, 103*, 104, 105, 106, 107, 108, 109*, 110, 111, 112*, 145*, 146, 147, 148, 149, 150, 180*, 181, 182, 183, 184, 185, 186, 187, 188, 189, 190, 191, 192*, 193, 194	15 sec
	1POC	1	2	26	202	1, 2, 3, 4, 5, 6, 9, 10, 11, 14, 15, 22, 23, 24, 51, 52, 54, 55, 76, 77, 78, 79, 81, 82, 83, 85, 86, 89, 90, 91, 92	12*, 43*, 82	15 sec
	2K5G	7	2	30	152	67, 68, 71, 72, 73, 74, 75, 76, 77, 78, 79, 80, 81, 82, 83, 84, 85, 86, 143, 147, 148, 166, 167, 168, 169, 170, 171, 172, 173, 174, 175, 176, 177, 178, 179, 180, 181	73, 81, 82, 83, 84, 85, 86, 109*, 141*	15 sec

	2R55	29	6	0	196	81, 84, 104, 105, 106, 107, 108, 109, 110, 111, 112, 113, 145, 146, 147, 148, 149, 182, 183, 184, 185, 186, 187, 188, 189, 190, 191, 192, 195	81, 84, 104, 105, 106, 107, 108, 109, 110, 111, 112, 113, 114*, 144*, 145, 146, 147, 148, 149, 150*, 181*, 182, 183, 184, 185, 186, 187, 188, 189, 190, 191, 192, 195, 196*, 198*	15 sec

Table S7: The performance of DREAMM, PPM3, MODA, PMIpred, and the MLP models trained on ProtTrans embeddings, and ESM embeddings, for four proteins of the test set, collectively. The model with the best F_1 and MCC scores are in bold.

Model	TP	FP	FN	TN	F_1 score	MCC score
DREAMM[27]	15	7	107	738	0.21	0.26
PPM3[28]	9	6	113	739	0.13	0.18
MODA[29]	23	2	99	743	0.31	0.39
PMIPred[30]	3	13	119	732	0.04	0.02
MLP ProtTrans	65	11	57	734	**0.66**	**0.64**
MLP ESM	63	16	59	729	0.63	0.6

Table S8: The peripheral membrane proteins of the extra test set, their PDB ID, their experimentally known membrane-interacting amino acids or regions, and the predictions of our MLP models. Amino acid numbering is consistent with the PDB structure.

Protein – PDB ID	Membrane-interacting regions / amino acids	ProtTrans MLP	ESM MLP
1c1z	L313, F315, W316	-	C215, N283
1coy	Around Amino acid M81	V275	-
1dvp	F173	-	R162, E170, F171, T174, N175, R176, K177, H178, Q195, G202, E204
1es6	L295, V298	L203	-
1ffj	L6, V7, P8, L9, F10, Y22, M24, F25, M26, V27, P30, V32, P33, V34, I39, L47, L48, V49	L6, V7, L9, A28	K4, A29, V32
1gyg	Y331, F334	N60, D216, E267, D269, A270, G271, D273, N294, G296, N297, M300, T301, Y331, T332, A333, F334, P335	S265, G266, D269, G271, D273, G296, D298, T301, K330, Y331, T332, A333, F334, D336
1h0a	L6, M10, I13, V14	D31, W33, D118, Q119	P32, P35, K117
1iaz	W112, Y113	-	S41, P81, R131
1joc	V1367, T1368, V1369	V1355, V1367, S1394, S1395, K1396, K1397	T1368, R1371, P1398
1nl1	F5, L6, V9	-	-
1oiz	F165, F169, I202, V206, M209	V201, I202, F203, H204, V206, M209, I210, P212, L214	K75, P173, K219

1pfo	W466, T490, L491	L462	A463
1s6x	F5, M6, W7, W27, V29, L30	-	-
1tqn	F-G loop and helices (218- 238) and A-anchor (44-47)	-	P45, F304
1vfy	L185, L186	E217	S173, K181, K182, K189, H190
2ayl	I74, W75, W77, L78, F88, F91, L92, W98, L99, F102	-	-
2ddr	W284, F285	T236, N237, N243, F244, P245, D246, S247, W279, V281, T282, W284, F285, Q286, K287	P26, G29, L172, Y293
2fnq	W413, F414, Y448, W449	R385, E386, H387, A388, G389, T390, D391, H415, N416, D417, E419, G445, G447, Y448, W449, D452, P453, I691	D384, E386, T390, D391, H415, N416, G446, G447, Y448, W449, D452, V479
2mh1	W22, P23, V24, L30, P31, V32	-	-
2p0m	Y15, F70, L71, W181, L195	I14, Y15, A16, K19, F70, L71, K72	A12, S13, I14, Y15, A16, G17, S18, R40, R43, H69, L71, K72, E73, D74, V101, G114, T116
3akm	Around amino acid K27	K16, F17, E19, K20, G22, V23, N24, I25, V26, K27, K29, D34, A54, F55, R56, N57, N71, A73, D74, G75, N98, N100, E120	K20, K27, D97, N98, G99
3fsn	F196, F200, I202, F264, L265, W268, L270, W271	A107, K355, A357, Q420, L439, A495	P56, F235, Y340

3iiq	W300, W310	F196, K213, W310	D142
3jw8	Around amino acids L179, L186	E96, S101, D102, L184, A213, E284	L179, V180, A213
3npe	The two parallel amphipathic helices (α1:85- 109, α2:222- 237)	D163, V165, A166, I215, L218, G220, I224, D240, E499, W501	E499, K503
3rzn	W142	Y81, N119, W142	-
3w7r	Region 31-68	P138, W362	E127, N129, R136, N145, R146, S151
4iap	R232, Q1237, G1238, F1239, K1240, K1241, L1257, N1258, H1260, N1261, Q1262, T1263, R1265	T1021, E1022, G1023, Y1024, N1055, Q1105, N1140, Q1141	-
4x08	W35	-	-
5bzz	Regions 260- 269, 327- 335	G20, E43, F206, G208, G209, T210, K267	R41, E43, P204, K237, H259, Q261, D268, E285, N329, K330, K332
5f0p	From chain C F103, F110	-	-
5hxw	I345, L347, L351, I352, F355	-	G86, K160
6bfg	Region 177- 215	P20, D168, K174, T339	R199, H200, T213, R225, Q278, L333

Table S9: The hyperparameters that were sampled for the LGBM classifier for each dataset using PDB sequences, the ranges that were searched in Optuna optimization, and the final best hyperparameters for each dataset.

Algorithm	Dataset	Hyperparameter ranges	Best hyperparameters identified by hyperparameter optimization
LGBM*	ProtTrans	Objective: binary,<br>Boosting type: gbdt,<br>Metric: binary_logloss<br>1e-4 ≤ Learning rate ≤ 1e-1<br>1 ≤ Max depth ≤ 32, step = 2<br>20 ≤ Number of leaves ≤ 100<br>0.5 ≤ Feature Fraction ≤ 1<br>0.5 ≤ Bagging Fraction ≤ 1<br>1 ≤ Bagging Freq ≤ 10<br>0 ≤ Lambda 1 ≤ 1<br>0 ≤ Lambda 2 ≤ 1<br>100 ≤ Number of iterations ≤ 2000, step = 100<br>1.1 ≤ Scale pos weight ≤ 11.1	learning_rate: 0.062302206771792<br>max_depth: 13<br>scale_pos_weight: 10.9757501531<br>num_leaves: 44<br>feature_fraction: 0.775041730621<br>bagging_fraction: 0.992966514496<br>bagging_freq: 8<br>lambda_l1: 0.5166450584607746<br>lambda_l2: 0.08760774771397549<br>num_iterations: 1500
	ESM		learning_rate: 0.050442985700119<br>max_depth: 11<br>scale_pos_weight: 10.3941974063<br>num_leaves: 48<br>feature_fraction: 0.811448655461<br>bagging_fraction: 0.631237526329<br>bagging_freq: 9<br>lambda_l1: 0.5139572446725574<br>lambda_l2: 0.7041685416885489<br>num_iterations: 2000

References

1. Cock, P. J. a *et al.* Biopython: freely available Python tools for compu... [Bioinformatics. 2009] - PubMed result. *Bioinformatics* **25**, (2009).
2. Vaswani, A. *et al.* Attention is all you need. in *Adv Neur In* vols 2017-December (2017).
3. Elnaggar, A. *et al.* ProtTrans: Towards Cracking the Language of Life's Code Through Self-Supervised Learning. *TPAMI / PAMI* **14**, (2021).
4. Elnaggar, A. *et al.* ProtTrans. *bioRxiv* **14**, (2020).
5. Rao, R. *et al.* Evaluating protein transfer learning with TAPE. in *Adv Neur In* vol. 32 (2019).
6. Rao, R., Meier, J., Sercu, T., Ovchinnikov, S. & Rives, A. Transformer protein language models are unsupervised structure learners. *bioRxiv* Preprint at (2020).
7. Rao, R. *et al.* MSA Transformer. *bioRxiv* (2021).
8. Raffel, C. *et al.* Exploring the limits of transfer learning with a unified text-to-text transformer. *J Mach Learn Res* **21**, (2020).
9. Clark, K., Luong, M.-T., Le, Q. v & Manning, C. D. ELECTRA: Pre-training Text Encoders as Discriminators Rather Than Generators. Preprint at (2020).
10. Devlin, J., Chang, M. W., Lee, K. & Toutanova, K. BERT: Pre-training of deep bidirectional transformers for language understanding. in *NAACL HLT 2019* vol. 1 (2019).
11. Lan, Z. *et al.* ALBERT: A Lite BERT for Self-supervised Learning of Language Representations. Preprint at (2019).
12. Dai, Z. *et al.* Transformer-XL: Attentive language models beyond a fixed-length context. in *ACL 2019* (2020).
13. Yang, Z. *et al.* XLNet: Generalized autoregressive pretraining for language understanding. in *Adv Neur In* vol. 32 (2019).
14. Lin, Z. *et al.* Evolutionary-scale prediction of atomic level protein structure with a language model. *Science (1979)* (2023).
15. Jumper, J. *et al.* Highly accurate protein structure prediction with AlphaFold. *Nature* **596**, (2021).

16. Baek, M. *et al.* Accurate prediction of protein structures and interactions using a three-track neural network. *Science (1979)* **373**, (2021).

17. Boughorbel, S., Jarray, F. & El-Anbari, M. Optimal classifier for imbalanced data using Matthews Correlation Coefficient metric. *PLoS One* **12**, (2017).

18. Tang, Y., Zhang, Y. Q. & Chawla, N. v. SVMs modeling for highly imbalanced classification. *IEEE Trans Syst Man Cybern* **39**, (2009).

19. Snoek, J., Larochelle, H. & Adams, R. P. Practical Bayesian optimization of machine learning algorithms. in *Adv Neur In* vol. 4 (2012).

20. Passos, D. & Mishra, P. A tutorial on automatic hyperparameter tuning of deep spectral modelling for regression and classification tasks. *Chemometr Intell Lab* vol. 223 Preprint at (2022).

21. Tubiana, T., Sillitoe, I., Orengo, C. & Reuter, N. Dissecting peripheral protein-membrane interfaces. *PLoS Comput Biol* (2022).

22. Costeira-Paulo, J. *et al.* Lipids Shape the Electron Acceptor-Binding Site of the Peripheral Membrane Protein Dihydroorotate Dehydrogenase. *Cell Chem. Biol.* **25**, (2018).

23. Nasr, M. L. *et al.* Membrane phospholipid bilayer as a determinant of monoacylglycerol lipase kinetic profile and conformational repertoire. *Prot. Sci.* **22**, (2013).

24. Kiser, P. D. *et al.* Structure of RPE65 isomerase in a lipidic matrix reveals roles for phospholipids and iron in catalysis. *PNAS* **109**, (2012).

25. Baylon, J. L., Lenov, I. L., Sligar, S. G. & Tajkhorshid, E. Characterizing the membrane-bound state of cytochrome P450 3A4: Structure, depth of insertion, and orientation. *J. Am. Chem. Soc.* **135**, (2013).

26. Hoi, J. J. *et al.* Solution structure and lipid membrane partitioning of VSTx1, an inhibitor of the KvAP potassium channel. *Biochem.* **44**, (2005).

27. Chatzigoulas, A. & Cournia, Z. DREAMM: a web-based server for drugging protein-membrane interfaces as a novel workflow for targeted drug design. *Bioinform* **38**, 5449–5451 (2022).

28. Lomize, A. L., Todd, S. C. & Pogozheva, I. D. Spatial arrangement of proteins in planar and curved membranes by PPM 3.0. *Protein Sci* **31**, (2022).

29. Kufareva, I. *et al.* Discovery of novel membrane binding structures and functions. *Biochem Cell Biol* **92**, (2014).

30. Hilten, N. van *et al.* PMIpred: A physics-informed web server for quantitative Protein-Membrane Interaction prediction. *bioRxiv* 2023.04.10.536211 (2023).
